# Toxicity of *Tetradium ruticarpum*: Subacute Toxicity Assessment and Metabolomic Identification of Relevant Biomarkers

**DOI:** 10.3389/fphar.2022.803855

**Published:** 2022-02-28

**Authors:** Qiyuan Shan, Gang Tian, Xin Han, Hui Hui, Mai Yamamoto, Min Hao, Jingwei Wang, Kuilong Wang, Xianan Sang, Luping Qin, Guanqun Chen, Gang Cao

**Affiliations:** ^1^ School of Pharmaceutical Science, Zhejiang Chinese Medical University, Hangzhou, China; ^2^ Department of Agricultural, Food and Nutritional Science, University of Alberta, Edmonton, AB, Canada; ^3^ Department of Biological Sciences, University of Alberta, Edmonton, AB, Canada; ^4^ The Public Platform of Medical Research Center, Academy of Chinese Medical Science, Zhejiang Chinese Medical University, Hangzhou, China

**Keywords:** *Tetradium ruticarpum*, metabolomics, toxicity, toxicity attenuation, toxicity biomarkers

## Abstract

*Tetradium ruticarpum* (TR) is widely used in Asia to treat gastrointestinal disorders and pain. Stir-frying with licorice aqueous extract is a traditional processing procedure of TR formed in a long-term practice and performed before clinical application, and believed to reduce TR’s toxicity. However, its toxicity and possible toxicity attenuation approach are yet to be well investigated. Subacute toxicity and metabolomics studies were conducted to help understand the toxicity of TR. The subacute toxicity assessment indicated that 3 fold of the recommended therapeutic dose of TR did not show obvious subacute toxicity in rats. Although an extremely high dose (i.e., 60 fold of the recommended dose) may cause toxicity in rats, it reversed to normal after 2 weeks of recovery. Hepatocellular injury was the major toxic phenotype of TR-induced liver damage, indicating as aspartate aminotransferase (AST) and liver index increasing, with histopathologic findings as local hepatocyte necrosis, focal inflammatory cell infiltration, slightly bile duct hyperplasia, and partial hepatocyte vacuolation. Moreover, we evaluated the impact of processing in toxicity. TR processed with licorice could effectively reduce drug-induced toxicity, which is a valuable step in TR pretreatment before clinical application. Metabolomics profiling revealed that primary bile acid biosynthesis, steroid biosynthesis, and arachidonic acid metabolism were mainly involved in profiling the toxicity metabolic regulatory network. The processing procedure could back-regulate these three pathways, and may be in an Aryl hydrocarbon Receptor (AhR) dependent manner to alleviate the metabolic perturbations induced by TR. 7α-hydroxycholesterol, calcitriol, and taurocholic acid were screened and validated as the toxicity biomarkers of TR for potential clinical translation. Overall, the extensive subacute toxicity evaluation and metabolomic analysis would not only expand knowledge of the toxicity mechanisms of TR, but also provide scientific insight of traditional processing theory, and support clinical rational use of TR.

## Introduction

The fruits of *Tetradium ruticarpum* (A. Juss.) T. G. Hartley (herein called “TR”), a common medicinal plant product in traditional Chinese medicine and Japanese Kampo, has been used for treating headache, vomiting, and gastrointestinal disorders in oriental history (eFlora of China, 2008; Commission of Chinese Pharmacopeia, 2020). The chemical compounds isolated from TR are mainly alkaloids [including evodiamine (EVO), rutaecarpine (RUT), dehydroevodiamine (DHE), and quinolone alkaloids], terpenoids, flavonoids, phenolic acids, and volatile oils (Li and Wang, 2020). Pharmacological studies revealed that the extracts of TR and their bioactive components have remarkable potential therapeutic values in treating tumors, cardiovascular disorders, antimicrobials, Alzheimer’s disease, and obesity in clinical practice (Park et al., 1996; Ono et al., 2011; Pan et al., 2014; Gavaraskar et al., 2015; Tian et al., 2019; Li and Wang, 2020).

Although TR’s medicinal value has been widely recognized, the potential risks of its toxicity have always been a concern in clinical application. The aqueous, ethanol, and chloroform extracts of TR, as well as their volatile oils, have certain levels of toxicity (Zhang et al., 2011; Cai et al., 2014; Liang J et al., 2017). Aqueous extract of TR at doses of 10–30 g/kg for 21 continuous days in mice led to the toxicity by the increase of inflammatory factors (Zhou et al., 2013), while the no–observed–adverse–effect–level of aqueous extract of TR in rats was reported to be greater than 2 g/kg (Kim et al., 2014). We previously measured that the exact 50% lethal dose (LD_50_) and 5% lethal dose (LD_5_) of aqueous and 70% ethanol extracts of TR in mice are much higher than the clinical recommended dose [2–5 g per day in humans, the dose conversion method was referred by the FDA Guidance for Industry (https://www.fda.gov/media/72309/download)] (Shan Q et al., 2020). With limited toxicity assessment of TR, it is urgent to assess the toxicity potential of TR under a long-time exposure with a wide dose range, as well as elucidate the possible mechanisms of toxicity.

TR is usually preprocessed before clinical application (Li and Wang, 2020; Shan QY et al., 2020). Stir-frying with licorice aqueous extract is one of the standard processing methods and formed in a long-term practice that may attenuate the acute toxicity and improve the efficacy of TR (Shan Q et al., 2020). However, whether the processing method could alleviate toxicity in the subacute timescale or influence the potential detoxification mechanisms is unclear. In addition, TR contains a set of complex, naturally derived components, and their efficacy or toxicity on the human body is likely much more complicated than that of a single agent, gene, or protein. Therefore, the action modes of TR would be ideally elucidated from a holistic perspective with methods such as untargeted metabolomics analysis and associated validation, which have been proven to be an effective method to achieve such goals (Johnson et al., 2016; Chong et al., 2018; Huan et al., 2018).

The aim of this study was to, 1) investigate the subacute toxicity of TR under 3 to 60 times of the clinic recommended doses, 2) evaluate the toxicity attenuation by the traditional processing procedure (stir-frying by licorice aqueous extract), 3) explore the metabolic toxicity and detoxification mechanism with transcript-level validation, and 4) identify potential biomarkers with subacute toxicity assessment. The results would not only expand our knowledge of the toxicity mechanisms of TR, but also provide scientific insight of traditional processing theory and support the clinical rational use of TR.

## Materials and Methods

### Materials and Reagents

Licorice roots (*Glycyrrhiza uralensis*, Inner Mongolia of China) and TR fruits (Yueyang, China) were purchased from Zhejiang University of Chinese Medicine Traditional Chinese Medicine Sliced Medicine Co., Ltd. (Hangzhou, China). All these plant materials were authenticated by Prof. Shuili Zhang (School of Pharmaceutical Sciences, Zhejiang Chinese Medical University). The voucher specimens of the licorice roots and TR fruits were deposited in the School of Pharmacy, Zhejiang Chinese Medical University (Nos. 20180903-gc and 20180819s, respectively). Reference standards were purchased from Chengdu Mansite Biotechnology Co., Ltd (Chengdu, China).

### Standard Processing Procedure, Identification, Quantification, and Extraction Preparation

Raw and processed TR were used in the study. The processed TR and their extracts were produced based on the previous method (Shan Q et al., 2020). In brief, 6 g of licorice pieces were used for processing 100 g of TR. Licorice pieces were immersed in water and boiled for 30 min. The extract was collected and fresh water was added to the licorice pieces and boiled again for the second extraction. The extractions were combined, concentrated, and filtered. The condensed licorice aqueous extract was added to 100 g of raw TR fruit for full absorption, which were then stir-fried until dry (No.20180819z).

The dried fruit of raw and processed TR (30 kg) were extracted with an 8-fold volume of 70% ethanol for 1 h, twice, under refluxing. The solvent was evaporated by a vacuum rotary system (IKA, Germany) and reduced to 3 g/ml (high dose). The extracts were diluted by distilled water to low (0.15 g/ml) and moderate (1.5 g/ml) doses before use.

The components of raw and processed TR were identified by ultra-high-performance liquid chromatography (UPLC)-quadruple time of flight mass spectrometry (QTOF-MS) (Shan Q et al., 2020) as shown in Supplementary Figure S1, and the main components were determined (Supplementary Table S1) by the method we previously established (Hui et al., 2021).

### Animals

Male and female Sprague-Dawley (SD) rats (180–220 g) were purchased from Shanghai Laboratory Animal Center (Shanghai, China) and acclimatized for 7 days in the housing condition of the specific pathogen-free environment (22 ± 1°C temperature, 50 ± 5% humidity, 12 h light/dark cycle, standard pellet diet, and water *ad libitum*) before the experiment. Animal care and use were conducted in accordance with the principles and guidelines of National Institutes of Health (National Research Council, 2011), and the experimental protocol was approved by the Animal Care and Use Committee of Zhejiang Chinese Medical University (No. 20180716-11).

### Experimental Design, Observation, and Sample Collection for Subacute Toxicity Study

The overall experimental design is shown in Supplementary Figure S2. The rats were orally administrated with TR extracts for the subacute toxicity study. One hundred SD rats were randomly divided into 10 groups (10 rats per group; 5 females and 5 males). The low, moderate, and high doses were selected as 1.5, 15, and 30 g/kg, respectively, which were approximately 3 times of the recommended human dose (the recommended human dose is 2–5 g per day, the conversion factor from human to rat is 6.2 according to the FDA Guidance for Industry https://www.fda.gov/media/72309/download), 30 fold (about one-tenth of LD_5_, raw TR: 145.6 g/kg; processed TR: 160 g/kg), and 60 fold (one-eighth of LD_50_, raw TR: 222.0 g/kg; processed TR: 230.4 g/kg), respectively (Shan Q et al., 2020). Among the 10 groups of rats, 6 groups of rats were treated with low-, moderate-, and high-dose raw or processed TR extracts by oral gavage for 28 consecutive days. Two recovery groups were treated with raw or processed TR (4-weeks high-dose administration), followed by 2 weeks of recovery. The control group and control-R group (the negative control for the recovery group) were treated with water.

The body weight, food, and water consumption of all rats were recorded weekly before and during the treatment using the methods reported in previous studies (Yang et al., 2019). Food and water consumption were calculated accordingly. At the end of the oral administration experiment, the rats were anesthetized with sodium pentobarbital at 0.06 g/kg (i.p.) and blood were collected from heart, and carbon dioxide was applied to sacrifice. Samples and organs were collected for hematological, biochemical, histological, molecular, and metabolomics analyses as described in the following sections.

### Hematological, Biochemical, and Histological Analyses

Approximately 2 ml of blood sample per rat was used in the hematological analysis (Upadhyay et al., 2019). Serum samples were used in the biochemical analysis of aspartate aminotransferase (AST), glucose (GLU), triglycerides (TG), creatine kinase (CK), and lactate dehydrogenase (LDH) based on reported methods (Upadhyay et al., 2019). Heart, liver, lung, kidney, and brain samples were fixed in 10% neutral buffered formalin for 24 h at room temperature and then subjected to standard procedures for hematoxylin-eosin (H&E) microscopic examination (Loha et al., 2019).

### Experimental Design and Sample Preparation for Metabolomics Analysis

Metabolomics analysis was conducted using serum samples to further study the dose-effect and time-course factors of the oral administration of raw and processed TR in rats (Supplementary Figure S2). For the dose-response analysis, serum samples were harvested from male rats in the subacute toxicity study as described above. For the analysis of time-course factor, another set of male SD rats were used. In brief, 90 male SD rats were randomly divided into nine groups (10 rats/group). One group served as the negative control, and six groups were treated with high-dose raw or processed TR (30 g/kg) for 1, 2 or 4 weeks. Two groups of rats were used as the recovery groups, which were treated with high-dose raw or processed TR for 4 weeks, followed by 14 days of clearance. At the end of the experiment, rats in all groups were anesthetized with sodium pentobarbital (0.06 g/kg, i.p.) for blood sample collection.

Blood samples were allowed to stand still for 30 min for coagulation and then centrifuged (3000 rpm, 10 min). Supernatants were collected and stored at −80°C. Next, samples were prepared for the metabolomics study. Serum sample (200 µl) was mixed with 400 µl of cold methanol and vortexed for 30 s to precipitate proteins. After centrifugation at 12,000 rpm for 15 min, 100 µl of the supernatant was transferred to sample vials with inserts for injection. A pooled quality control (QC) sample was generated by combining 10 µl of supernatant from each tested sample. The QC sample was used to equilibrate the analytical platform at the beginning and during the process (one injection per every 10 samples) (Dunn et al., 2012). All samples were kept at 4°C before injection.

### Liquid Chromatography Conditions and Mass Spectrometry Conditions

The serum samples were analyzed on a UPLC system coupled with a SYNAPT G2 QTOF high-definition MS system (Waters, Milford, MA, United States) and separated on a Acquity UPLC^®^ BEH C_18_ column (2.1 × 100 mm, 1.7 µm). The mobile phase was composed of solvent A (acetonitrile) and solvent B (deionized water containing 0.1% v/v formic acid). The detailed chromatography and MS conditions were set based on our previous work (Shan Q et al., 2020).

### Data Processing, Multivariate Analysis, and Biomarker Analysis

Raw data were acquired and preprocessed by MassLynx, UNIFI 1.7, and Progenesis QI (Waters, Milford, MA, United States). Data was collected in both ion modes and subjected to signal drift correction by StatTarget (https://www.bioconductor.org/packages/release/bioc/html/statTarget.html) using the suggested parameters (Luan et al., 2018). The converted data were then exported to MetaboAnlayst 4.0 for principal components analysis (PCA), partial least squares discriminant analysis (PLS-DA), orthogonal partial least-squares discrimination analysis (OPLS-DA), volcano plot analysis, and pathway analysis (Chong et al., 2018). A heatmap generation was produced by R (R Core Team, https://www.r-project.org). After normalization, potential biomarkers were selected according to the PLS-DA and volcano plot results with minor parameter adjustment [variable importance in projection (VIP) > 1, fold change (FC) > 2 or < 0.5, and *p* value after false discovery rate (FDR) < 0.05], and receiver operation characteristic (ROC) curve analysis was performed (Dunn et al., 2012; Chen et al., 2016; Di Guida et al., 2016). In the ROC analysis of individual biomarkers, the optimal threshold was determined by max (sensitivity + specificity), followed by the web-based software instruction. The potential biomarkers were compared with the chemical standards, filtered by interquantile range, normalized by median, log transformed (base 10), and then tested by multivariate ROC curve based exploratory analysis to explore, test, and cross-validate the biomarkers by three machine learning mathematical models [PLS-DA, Random Forest, and Linear Support vector machine (SVM)] using the biomarker analysis module in MetaboAnalyst 4.0 (Chong et al., 2018).

### Gene Expression Analysis with Quantitative Real-Time Polymerase Chain Reaction

Total RNA was extracted from liver tissue samples and reverse-transcribed to cDNA. QRT-PCR analysis was performed with SYBR Green PCR Master Mix (Roche, Germany) on QuantStudio PCR (Life technologies, United States). The primers synthesized by General Biosystems (Chuzhou, China) are listed in Supplementary Table S2. Gene expression levels were calculated using the 2^−△△Ct^ method (Schmittgen and Livak, 2008).

### Statistical Analysis

Results were expressed as mean ± standard derivation, unless otherwise specified. For group comparison of pharmacological effects, Dunnett T test and Dunnett’s T3 test were applied when the variances among the groups had homogeneity and had no homogeneity, respectively. All statistical analyses were performed with IBM SPSS statistics (IBM Corporation, Armonk, NY) and the graphs were produced by GraphPad PRISM 8.0 (GraphPad Software, Inc., San Diego, CA, United States).

## Results

### Subacute Toxicity of TR

Male and female rats were treated with a broad dose range of raw and processed TR extracts, which were approximately 3, 30, and 60 fold of the recommended human dose, for 28 consecutive days to test the possible subacute toxicity of TR. Two groups were also treated with high-dose raw and processed TR for 4 weeks and placed in recovery for 2 weeks to test the recovery of rats after TR treatment.

### Histopathology

**FIGURE 1:**
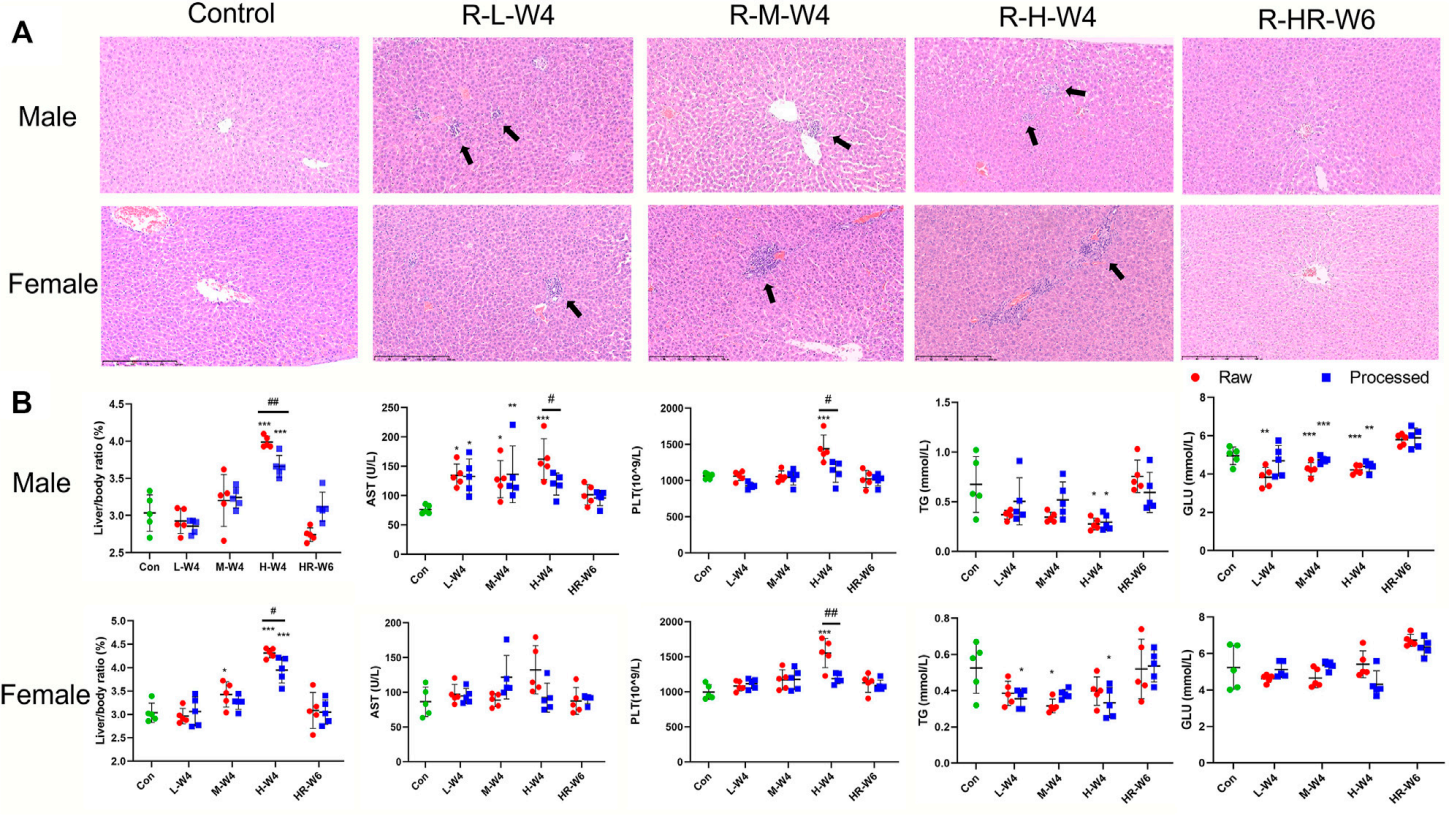
TR-induced hepatotoxicity in rats. **(A)** Typical liver histopathological results (×100) of male and female rats in the raw TR groups (treated with raw TR for 28 days; L, low dose; M, medium dose; H, high dose), recovery group (HR: treated with high-dose TR for 28 days and then 14-days recovery), and control group. **(B)** Typical indicators of alterations in blood and serum biochemistry after treatment with raw and processed TR. Data are expressed as means ± standard derivation (n = 5). *p < 0.05, **p < 0.01, ***p < 0.001 compared with the control group.

Microscopic inspection was conducted to evaluate the changes in the main organs of the male and female rats. As shown in Figure 1A, local hepatocyte necrosis, focal inflammatory cell infiltration, mild bile duct hyperplasia, and partial hepatocyte vacuolation lesions were found in the liver of male and female rats treated with raw and processed TR, but the rats treated with processed TR had some extent of toxicity attenuation in the liver (Supplementary Figure S3A). No other systemic changes in the other main organs of the rats treated with high-dose TR were observed (Supplementary Figure S3B).

### Body Weight, Food Consumption, and Water Intake in the Subacute Toxicity Study

The body weight, food consumption, and water intake of rats in different treatment groups were assessed every week for general toxicity evaluation in the subacute toxicity study. Male rats treated with high-dose of raw TR had a remarkable weight gain reduction at days 21 and 28 compared with those in the control group. The female recovery group, after high dose processed TR treatment at day 35, also had weight gain reduction. However, this phenomenon may be caused by individual differences, because no other weight gain reduction was observed before or after day 35. Besides, the female groups treated with high-dose raw and processed TR drank more water and no other significant changes in food and water intake were found (Supplementary Figure S4, Supplementary Table S3). No mortality was observed in any group throughout the experiment.

### Organ Coefficient

Organ coefficient is one of the most sensitive toxicity parameters, and remarkable changes may reveal early damage even before morphological changes happen (Piao et al., 2013). As shown in Figure 1B and Supplementary Table S4, the relative liver/weight ratio (liver index) in both males and females administrated with raw TR at high dose were significantly increased when compared with those of control (*p* < 0.001). Only the liver index of male rats elevated when administered with high-dose of processed TR, but attenuation was observed when compared with the raw TR-treated groups (*p* < 0.05). Therefore, although male and female rats had increased liver index after the oral administration of high-doses of TR, processed TR had reduced toxicity of the liver in male and female rats compared with raw TR. The relative weights of the kidney, testicle, and epididymis of rats treated with high-dose raw TR significantly increased (*p* < 0.05, *p* < 0.05, and *p* < 0.01, respectively) and processed TR treatment also had reduced toxicity in these organs of male rats when compared with those of raw TR (Supplementary Table S4). In addition, the liver index of rats treated with high-dose TR returned to normal after 2 weeks of recovery (Figure 1, Supplementary Table S4).

### Hematology and Serum Biochemistry

Blood samples of male and female rats treated with TR were collected and assessed for hematology and serum biochemical analysis. As shown in Figure 1B, Supplementary Tables S5, S6, high-dose raw TR treatment resulted in the remarkable elevation of platelet (PLT) levels in both female and male groups, whereas those of processed TR did not lead to a PLT increase. Mean corpuscular hemoglobin concentration (MCHC) remarkably decreased in female rats, whereas plateletcrit (PCT) remarkably increased in male and female rats. Another parameter affected by TR was hemoglobin (HGB), which decreased in the female group treated with high-dose -raw TR. Other parameters, such as eosinophil percentage (EOS) and mean corpuscular hemoglobin (MCH), were within normal laboratory ranges, which indicated that no relative histopathological changes occurred (Giknis and Clifford, 2008). Interestingly, although the high dose is 60 times the recommended clinical amount, most of these disturbed parameters returned to normal after the recovery period. Together, although high doses of TR, especially raw TR, could introduce toxicity to the blood system with 28 days of administration, its toxicity can be reversed after the withdrawal of TR treatment.

AST, CK, and LDH levels increased, whereas ALP, GLU, and TG values decreased in the male groups treated with raw TR at different doses when compared with those of control (Figure 1B and Supplementary Table S6). High-dose processed TR did not cause AST elevation in the female and male groups, which indicated that the processing procedure could be a potential solution to reduce TR’s liver toxicity. Similarly, the elevation of CK and LDH in male rats treated with processed TR were lower than those caused by raw TR treatment. A reduction in TG was also observed in the processed TR-treated female and male rat groups, which demonstrated that treating with processed TR has equal or greater TG decreasing effects when compared with those of raw TR. In addition, most of the affected indicators were ameliorated after recovery.

### Metabolomics Analysis of TR Toxicity

A metabolomics approach was performed to landscape the broad toxic impact of TR treatment to rats under high-dose exposure, and the key enzymes and genes those may regulate the biosynthesis or transformation of metabolites in the most disturbed pathways were validated. The screened potential biomarkers were further evaluated by dose–effect and time-course group data with reference standard confirmation and then tested and cross-validated by training and prediction data sets. The results provided sensitive and specific potential biomarkers for TR toxicity prevention, diagnosis, and treatment.

### TR-Induced Toxic Metabolic Profiles

**FIGURE 2:**
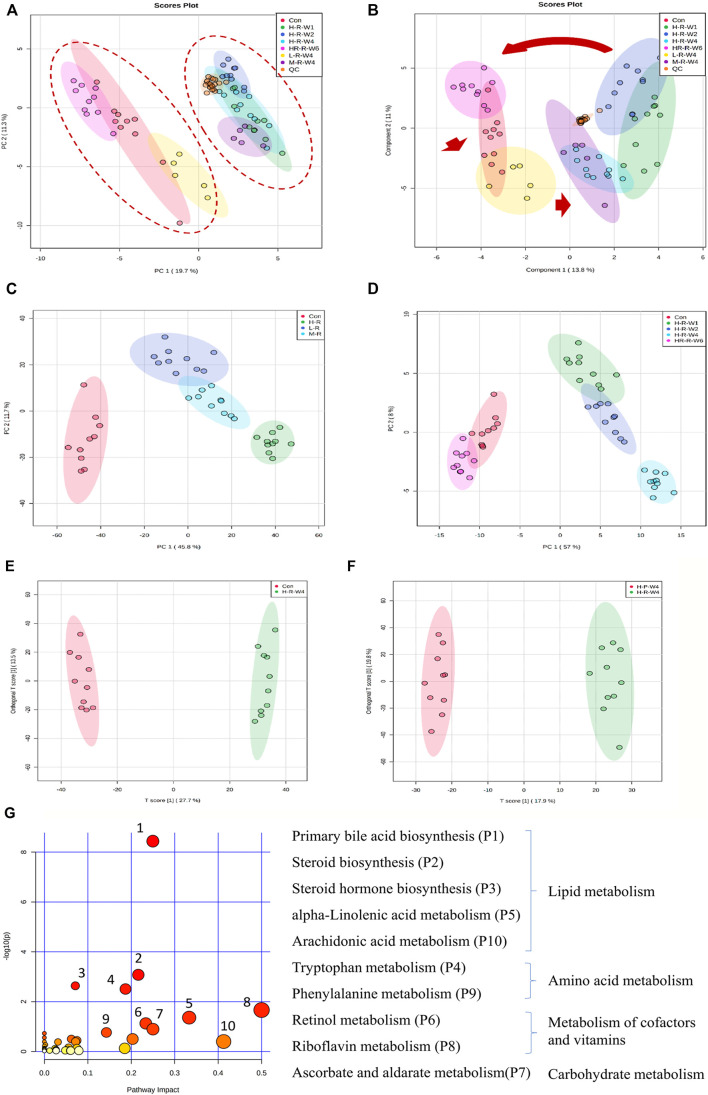
Clustering profile of rat serum samples after TR treatment in the positive ion mode. **(A)** PCA score plot and **(B)** sPLS-DA plot of male rats in the raw TR groups (treated with raw TR for 28 days; L, low dose; M, medium dose; H, high dose), recovery group (HR: 28 days of high-dose TR and followed by 14-days recovery), control group and QC sample; **(C)** dose–effect clustering results and **(D)** time–course clustering results of raw TR groups; **(E)** control group vs. high-dose raw TR treatment group for 28 days; **(F)** raw TR group vs. processed TR group after treatment for 28 days; **(G)** metabolic pathway analysis of high-dose TR in rats after 28 days of treatment.

A total of 14,854 and 11,879 peaks were detected in the serum samples under positive and negative modes, respectively. After whole groups were subjected to signal drift correction (detailed in Methods), 5,595 and 5,643 variables were retained in positive and negative ion modes. All groups of rat serum samples under both positive (Figure 2A) and negative ion mode (Supplementary Figure S5A) were analyzed to identify the metabolic perturbations among different treatment groups. The PCA plot is unsupervised and provides an informative first look at the relationships among multiple groups. The PLS-DA is supervised and can provide the detailed information of discriminations among groups. OPLS-DA is suitable for comparison between two groups and could also provide the most important variables which could explain the difference between groups (Blaise et al., 2021). Figure 2A, Supplementary Figure S5A are the PCA plots of general clustering profiles. The figures indicate the distinct separations of serum samples treated with different doses of TR extracts. The two major groups are clustered separately and the QC groups are clustered tightly in the center. Figure 2B, Supplementary Figure S5B are PLS-DA plots and show the dynamic progress profiles of dose–effect and time–course factors. The figures indicate a clear dose- and time - accumulated developing cycle. Figures 2C,D, Supplementary Figures S5C,D are PCA plots and Figures 2E,F, Supplementary Figures S5E,F are OPLS-DA plots.

### Effect of Dose on the Metabolic Perturbation

Metabolic changes that may be caused by dose, low-, moderate-, and high-dose of raw TR treated to rats were studied. As shown in Figure 2C and Supplementary Figure S5C, clusters of dose response in rats were clearly identified. The low-dose group was closer to the control, whereas the moderate- and high-dose groups were clustered together. The results indicate that the metabolic perturbations caused by TR-induced toxicity are dose dependent.

### Time—Course Insights into the Metabolic Perturbation

Based on the dose–effect results, high–dose TR was chosen to conduct a time–course study of TR–induced toxicity. As shown in Figure 2D and Supplementary Figure S5D, the PCA plots show that the groups clustered by treatment time clearly. The recovery group was clustered with the controls, whereas the 1-, 2-, and 4-week administration groups were clustered on the other side. Compared with the controls, the group treated for 4 weeks had the largest perturbations at the metabolic level. Moreover, the clustering result of the recovery group indicated that metabolic disturbance was reversible even in the treatment with 60 fold of the clinically recommended dose. These metabolic observations are in accordance with the serum AST level and liver/body weight alterations (Figure 1B).

### Metabolic Disturbances Caused by TR Toxicity, and Identification of Potential Toxic Metabolites

Global profiling of serum samples revealed the remarkable metabolic disturbances in the rats treated with raw TR for 28 days (Figure 2 and Supplementary Figure S5). OPLS-DA plots (Figure 2E and Supplementary Figure S5E) and related statistical analysis show the detailed information of TR toxicity. A total of 499 (positive) and 545 (negative) peaks were filtered out in positive and negative modes, respectively, based on the OPLS-DA model and volcano plot analysis of the control group versus the high-dose raw TR group treated for 28 days (VIP value > 1, FC > 2 or < 0.5, adjusted *p* < 0.05; Table 1). These potential features were further annotated by standard identification procedure, in which the accurate molecular weight, retention time, and MS/MS fragments were compared with the Human Metabolome Database (HMDB), MassBank, Kyoto Encyclopedia of Genes and Genomes (KEGG), and literature records. Sixty-three metabolites were identified under both ion modes (Table 1). Most of them were arachidonic acid and its derivates, bile acid, and cholesterol derivates. Based on the identified metabolites, the pathway analysis by MetaboAnalyst revealed that TR administration resulted in distinct shifts at the metabolic level. Lipid metabolism, amino acid metabolism, metabolism of cofactors and vitamins, and carbohydrate metabolism were perturbed by TR challenging (Figure 2G). Specifically, the perturbed pathways are primary bile acid biosynthesis (P1), steroid biosynthesis (P2), steroid hormone biosynthesis (P3), and tryptophan metabolism pathways (P4). Half of the top 10 perturbed pathways belong to lipid metabolism according to KEGG classification (Figure 2G).

**TABLE 1:** Annotated metabolites detected in the rat serum samples after TR administration for 28 days.

No.	Annotated metabolites	VIP	FC	Adjusted P value	AUC	KEGG ID
1	Prostaglandin G2	4.87	3.63	2.62E-03	0.92	C05956
2	p-Coumaraldehyde	4.49	44.09	2.66E-14	1	C05608
3	Dihydrotestosterone	4.07	2.31	1.59E-04	0.98	C03917
4	Arachidonic acid	3.10	2.11	1.23E-03	0.93	C00219
5	7-Hydroxycoumarin	3.10	33.23	7.76E-13	1	C09315
6	Dihomo-gamma-linolenic acid	2.97	2.42	5.63E-04	0.94	C03242
7	Hyodeoxycholic acid	2.84	0.32	1.88E-03	0.89	C15517
8	reduced Riboflavin	2.69	30.39	4.99E-08	1	C01007
9^a^	7α-Hydroxycholesterol	2.66	0.11	8.94E-09	1	C03594
10^a^	Taurocholic acid	2.63	6.69	1.42E-06	1	C05122
11^a^	Cholic acid	2.55	0.24	5.00E-04	0.92	C00695
12^a^	Chenodeoxycholic acid	2.48	0.40	2.23E-02	0.83	C02528
13	9-cis-Retinol	2.34	2.04	1.12E-02	0.84	C16682
14	22α-Hydroxy-campest-4-en-3-one	2.34	0.28	1.17E-04	0.97	C15796
15	5β-Cholestane-3α,7α,12α,26-tetrol	2.25	0.19	8.16E-08	1	C05446
16	4-Hydroxycoumarin	2.25	46.66	1.43E-10	1	C20414
17	Testosterone propionate	2.19	0.29	1.48E-03	0.89	C08158
18^a^	Cholesterol	2.02	0.06	1.60E-08	1	C00187
19	13,14-Dihydroretinol	2.01	2.48	2.58E-03	0.91	C15492
20	Fumaric acid	1.96	0.43	1.56E-03	0.93	C00122
21	Eicosapentaenoic acid	1.89	0.50	5.96E-03	0.9	C06428
22	Riboflavin	1.80	2.41	1.39E-09	1	C00255
23^a^	Calcitriol	1.67	0.18	6.03E-09	1	C01673
24	Ursodeoxycholic acid	1.67	0.10	2.32E-05	0.97	C07880
25	Stearidonic acid	1.67	0.21	2.33E-04	0.94	C16300
26	4-(2-Amino-3-hydroxyphenyl)-2,4-dioxobutanoic acid	1.65	11.37	6.51E-12	1	C05645
27	13S-Hydroperoxy-9Z,11E-octadecadienoic acid	1.65	0.28	6.23E-06	0.99	C04717
28	N4-Acetylaminobutanal	1.65	16.68	9.73E-11	1	C05936
29	Presqualene diphosphate	1.63	0.13	7.62E-05	0.95	C03428
30	5-Hydroxyindoleacetic acid	1.62	2.70	2.24E-08	1	C05635
31^a^	D-Xylose	1.61	28.72	3.59E-12	1	C00181
32	(25S)-26-Hydroxycholest-4-en-3-one	1.60	0.37	2.85E-06	0.99	C20143
33	Bilirubin	1.59	0.39	6.03E-05	0.98	C00486
34	2-Aminobenzoic acid	1.59	44.92	1.17E-11	1	C00108
35	Calcidiol	1.56	0.30	9.42E-05	0.99	C01561
36	3α,7α,12α-Trihydroxy-5β-cholestan-26-al	1.51	0.46	2.97E-03	0.88	C01301
37	Phenylacetaldehyde	1.48	141.73	1.43E-12	1	C00601
38	Pipecolic acid	1.48	11.90	7.59E-12	1	C00408
39	(R)-3-Hydroxybutanoate	1.45	9.57	1.47E-14	1	C01089
40	Cinnavalininate	1.45	0.13	2.80E-06	1	C05640
41	5-Methoxyindoleacetic acid	1.44	2.31	6.61E-04	0.94	C05660
42	7α-Hydroxy-5β-cholestan-3-one	1.34	0.27	8.12E-09	1	C05451
43	Imidazolone	1.28	0.29	9.72E-08	1	C06195
44	16α-Hydroxydehydroisoandrosterone	1.25	4.78	4.42E-06	1	C05139
45	D-Ribose 5-phosphate	1.25	6.00	1.13E-10	1	C00117
46	7α-Hydroxy-3-oxo-4-cholestenoate	1.19	0.20	3.56E-03	0.94	C17337
47	Oxidized glutathione	1.19	2.60	1.84E-03	0.92	C00127
48	D-Glucuronolactone	1.19	10.37	4.71E-07	1	C02670
49^a^	S-Adenosylhomocysteine	1.17	5.66	9.84E-11	1	C00021
50^a^	Vitamin D3	1.17	0.13	4.86E-06	0.99	C05443
51	Phenylacetylglycine	1.16	0.35	8.35E-04	0.9	C05598
52	Cholesterol sulfate	1.15	0.11	2.26E-06	0.99	C18043
53	Lathosterol	1.14	0.23	7.10E-06	0.99	C01189
54	Etiocholanolone	1.12	0.41	6.91E-06	1	C04373
55	3α,7α-Dihydroxy-5β-cholestanate	1.11	0.10	2.32E-03	0.94	C04554
56	11-cis Retinol	1.09	2.01	8.19E-03	0.89	C00899
57	Etiocholanedione	1.08	2.4	4.14E-05	1	C03772
58	Leukotriene A4	1.08	2.16	2.76E-04	0.96	C00909
59	Vanylglycol	1.08	18.42	1.23E-12	1	C05594
60	Squalene	1.04	0.17	5.66E-05	0.96	C00751
61	Calcitetrol	1.02	0.19	1.34E-12	1	C18231
62	Glycocholic acid	1.01	0.18	2.58E-06	1	C01921
63	D-Xylulose	1.00	3.99	1.71E-11	1	C00310

a Compared to reference standard.

### Influence of TR-Induced Toxicity and Toxicity Attenuation on Metabolic Pathways at the Gene Transcription Level

**FIGURE 3:**
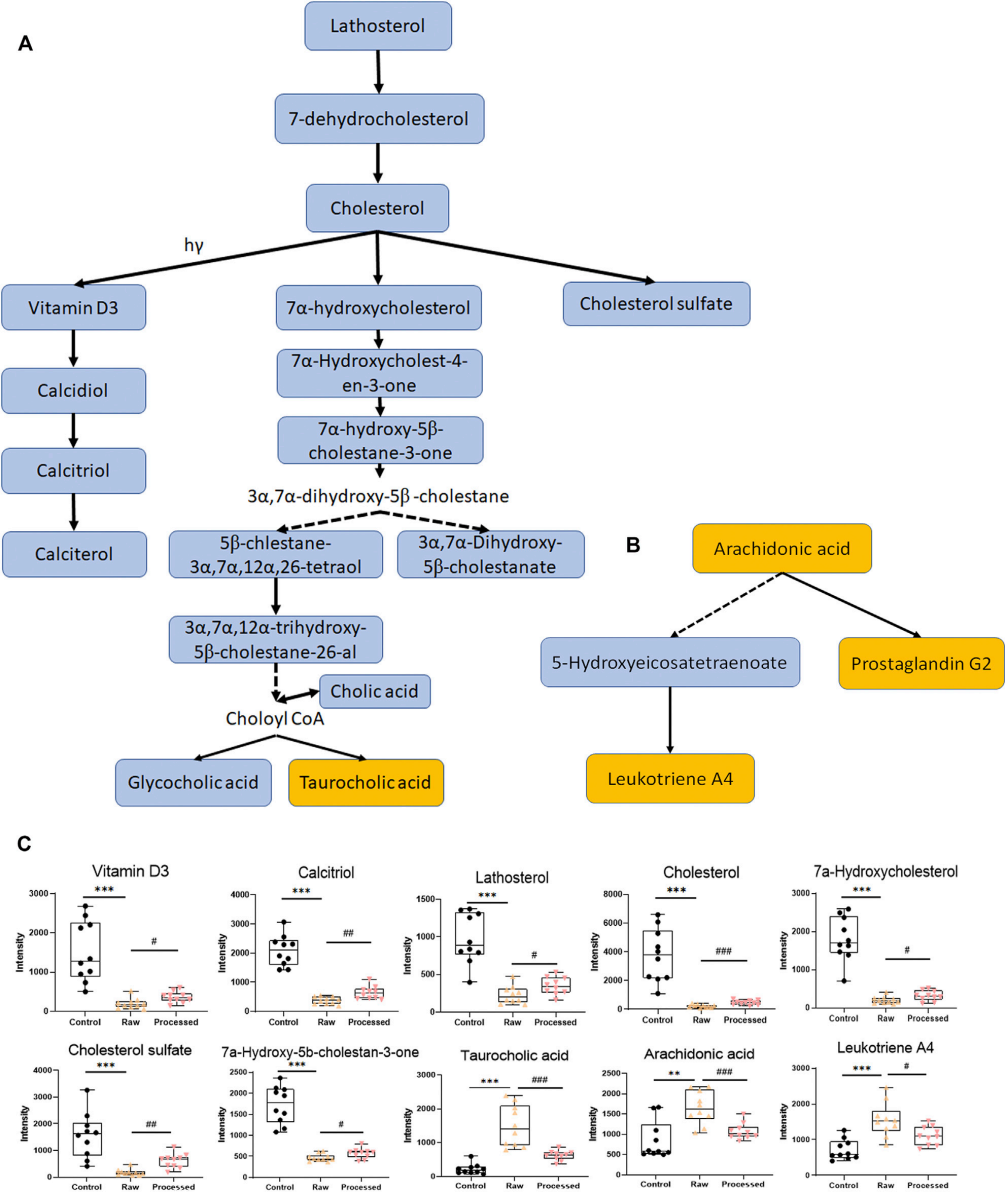
Typical disturbed pathways and relative intensity patterns of metabolites after 4 weeks of high-dose raw TR and processed TR administration. **(A)** Steroid biosynthesis pathway and primary bile acid biosynthesis pathway, **(B)** arachidonic acid metabolism, **(C)** relative intensity of typical disturbed metabolites. Data were expressed as means ± standard error of the mean (n = 10). *p < 0.05, **p < 0.01, and ***p < 0.001 for the comparison of the raw TR and control groups. ^#^ *p* < 0.05, ^##^ *p* < 0.01, and ^###^ *p* < 0.001 for the comparison of the processed TR and raw TR groups. Upregulated pathways are labeled in yellow, and downregulated pathways are labeled in blue.

**FIGURE 4:**
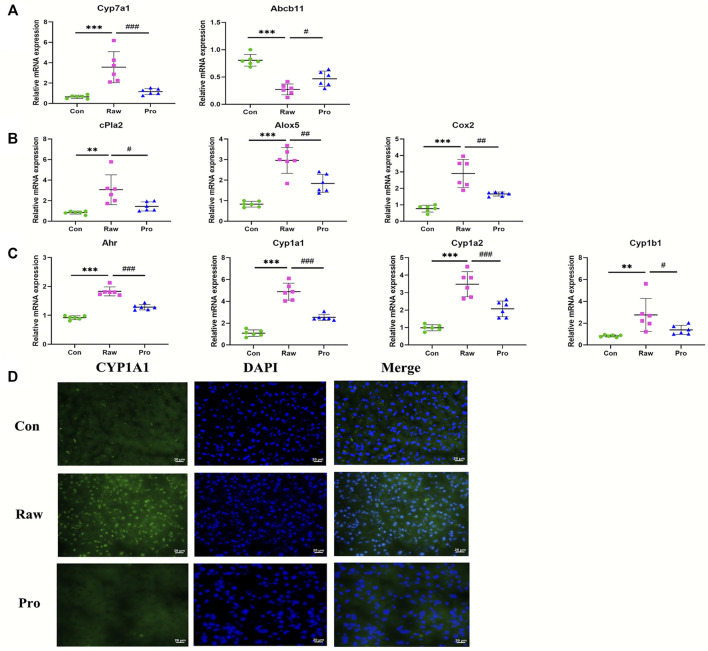
RT-qPCR analysis of liver mRNA expression in rats treated with 70% ethanol extract of TR. **(A)** Primary bile acid biosynthesis rate-limiting enzyme *Cyp7a1* and biliary salt excretion pump gene *Abcb11*; **(B)** arachidonic acid metabolic pathway activation-related genes: *cPla2*, *Alox5*, and *Cox2*; **(C)** *Ahr* and *Ahr* target genes: *Cyp1a1*, *Cyp1a2*, and *Cyp1b1.* Data are expressed as means ± standard error of the mean (n = 6). *p < 0.05, **p < 0.01, and ***p < 0.001 for the comparison of the raw TR and control groups. ^#^ *p* < 0.05, ^##^ *p* < 0.01, and ^###^ *p* < 0.001 for the comparison between the processed TR and raw TR groups. **(D)** Nuclear translocation of the *Ahr* target gene, *Cyp1a1*.

In the subacute toxicity study, we found that the processing method attenuated TR toxicity in histopathological (Figure 1A) and physiological conditions (Figure 1B). We further explored at the metabolic level to find clues for the mode of action of the toxicity attenuation caused by TR processing. We compared the 28-day, high-dose raw, and processed TR treatment groups (Figure 2F) and focused on the metabolites in the lipid metabolism pathways (primary bile acid biosynthesis, steroids biosynthesis pathway, and arachidonic acid metabolism). Interestingly, processed TR treatment alleviated toxicity in the metabolic level (Figure 3). When we focused on the main metabolites in lipid metabolism pathways, we found that the contents of the perturbed metabolites, such as primary bile acid (taurocholic acid), steroids (cholesterol, lathosterol, vitamin D3, calcidiol, and calcitriol), and inflammatory factors (arachidonic acid and leukotriene A4) were remarkably back-regulated when treated with processed TR. The expression of key genes in the pathways were analyzed to test if the toxicity and toxicity attenuation mechanisms were also regulated at the transcription level. As shown in Figure 4, the rate-limiting enzyme of bile acid biosynthesis, *Cyp7a1,* increased in the transcript level, whereas the biliary excretion of bile acid enzyme, *Abcb11* (or bile salt export pump BSEP), decreased considerably. The reduced *Acbc11* transcription level, linked with less steatosis and more inflammation after raw TR treatment, was in accordance with previous report (Fuchs et al., 2020). The transcription levels of pro-inflammatory factors, *cPla2*, *Alox5*, and *Cox2*, also increased and these key enzymes are involved in leukotriene biosynthesis and liver inflammation or injury (Enyedi et al., 2016). All these disturbed genes at transcript level were back regulated when treated with processed TR. The results revealed the possible mode of toxicity attenuation via these three pathways.

### Biomarker Validation and Pattern Analysis

**FIGURE 5:**
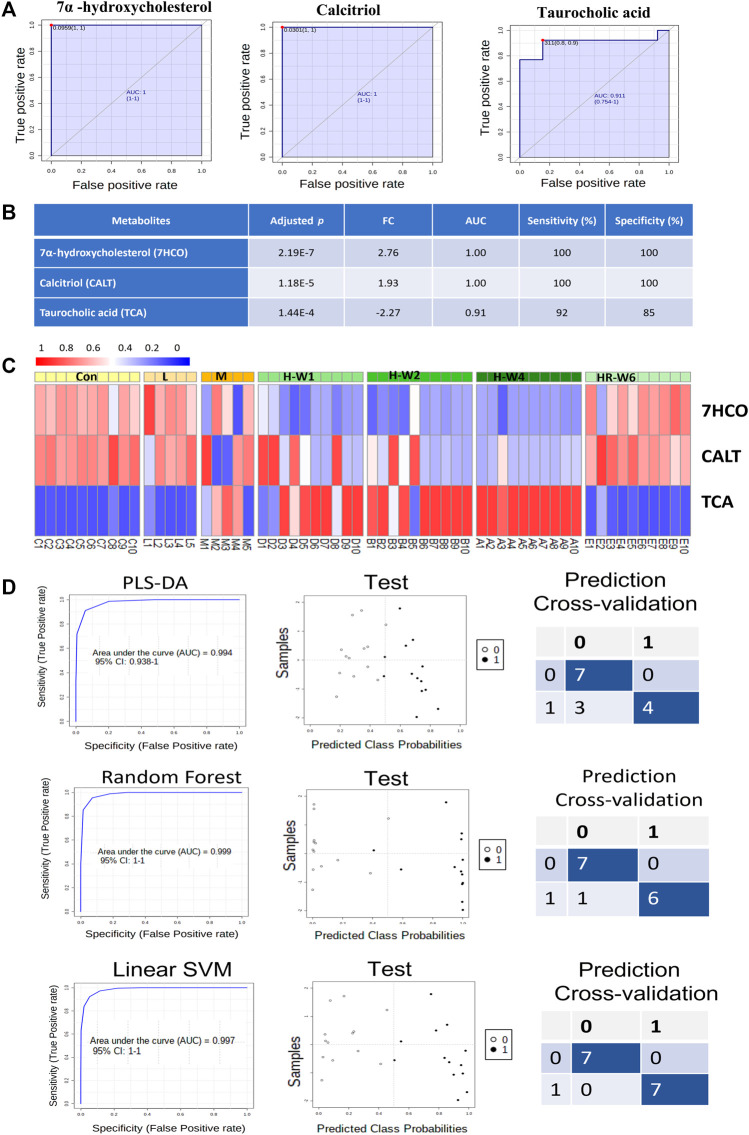
Validation of potential biomarkers of TR-induced toxicity. **(A)** ROC curves of the three biomarkers. **(B)** Adjusted *p* values, FC, AUC, sensitivity, and specificity of the three biomarkers. **(C)** Heatmap of the three biomarkers in the dose–effect and time–course groups after TR administration. **(D)** Test and prediction results of the models built with the three biomarkers by three machine learning mathematic algorithms: PLS-DA, Random Forest, and Linear SVM.

Based on the identified potential toxicity metabolites, receiver operation characteristic (ROC) curve analysis (the optimal threshold was determined by max (sensitivity + specificity), followed by web-based software instruction, was performed, and valuable features with area under the ROC curve (AUC) values larger than 0.8 were screened. The screened potential toxicity biomarkers were further validated in male groups to observe for histopathological, physiological, and metabolic progress patterns with dose and time dependence. Afterward, only the performance of three biomarkers were fitted with all these factors (Figures 5A–C). As shown in Figures 5A,B, the three metabolites (7α-hydroxycholesterol, calcitriol, and taurocholic acid) can serve as sensitive and specific potential biomarkers for TR-induced toxicity. All these potential markers are involved in lipid metabolism and show dose-dependent changes after TR administration (Figure 5C). The steroid-related metabolites (7α-hydroxycholesterol and calcitriol) remarkably decreased after 7 days of treatment and remained at a low level until withdrawal. The recovery group demonstrated a reverted pattern when compared with the high-dose and TR treatment groups. The third metabolite, taurocholic acid, had an opposite trend during administration compared with the other biomarkers (Figure 5C). The potential toxicity biomarkers were compared with chemical standards, filtered by interquantile range, normalized by median, log transformed (base 10), and applied in multivariate ROC curve-based exploratory analysis to explore, test, and cross-validate the results by three machine learning mathematic models (PLS-DA, Random Forest, and Linear SVM) through the biomarker analysis module in MetaboAnalyst 4.0 (Chong et al., 2018). The whole metabolomics data set size of the control group, high-dose recovery group, and three raw TR treatment groups was 60 (we excluded the processed TR treatment groups, because we could not explain how much the toxicity attenuation effect would be at the metabolic level). We chose the control group and 4-week high-dose TR treatment group to build the prediction models using the three biomarkers with PLS-DA, Random Forest, and Linear SVM modeling methods (with 20 samples). The models were then run on 2/3 of the left-out samples as the test data set (26 samples from the low-, moderate-, and high-dose TR treatments for 1 and 2 weeks and the recovery groups) to test whether we could obtain a clear classification through each prediction model. Cross-validation was then conducted on 1/3 of the left-out sample set (14 samples) according to the instruction of MetaboAnalyst (https://www.metaboanalyst.ca/docs/Faqs.xhtml). The Linear SVM method obtained the highest AUC and accuracy in the test and cross-validation prediction data sets (Figure 5D). Together, the results showed that the validated potential biomarkers were dose- and time-dependent, sensitive, and specific for TR administration even at the early stage, and the TR-induced metabolic perturbations were reversible after recovery. Therefore, the three biomarkers could be applied in TR-induced toxicity prevention, diagnosis, and treatment.

## Discussion

As a common medicinal plant product used in traditional Chinese medicine and Japanese Kampo, TR plays an important role in alternative traditional medicine. Investigating its toxicity, alleviation process, and identifying its biomarkers would contribute to TR’s clinical use; TR toxicity prevention, diagnosis, treatment; and TR’s mode of action. In this study, the toxicity of raw and processed TR at 3–60 fold of the recommended therapeutic dose (2–5 g per day in human), as well as its possible toxicity and toxicity attenuation mechanisms, were explored by toxicology and metabolomic analyses.

The hematological, biochemical, and histopathological results in rats treated with low-dose TR remain unchanged when compared with the control group (Figure 1; Supplementary Figure S3, and Supplementary Table S5, S6). The low dose used in the study is 3 fold of the recommended dose, but the subacute toxicity study revealed that this TR dose is relatively safe for 28-day administration.

We also used 30 and 60 fold of the clinically recommended TR dose in the experiments, although neither are currently used in clinics, to explore the potential *in vivo* toxicity and detoxification mechanisms and screen for biomarkers. The results showed that the liver was the main target organ of TR in both sexes under extreme high-dose TR administration (Figure 1 and Supplementary Figure S3A), which was consistent with other investigations (Li et al., 2013; Cai et al., 2014). Serum biochemistry and histopathology inspections indicated that hepatocellular injury was the major type of TR-induced liver damage, which was indicated by increased serum AST, decreased TG, liver local hepatocyte necrosis, neutrophil inflammatory infiltration, and hepatic bile duct hyperplasia (Figure 1B and Supplementary Table S6). Disturbances in serum TG and liver biliary pathology were further confirmed in the lipid and primary bile acid biosynthesis pathways, at metabolite level (7a-hydroxycholesterol, calcitriol, and taurocholic acid in Figure 3 and Table 1), and at transcriptional level (*Abcb11* and *Cyp7a1* in Figure 4).

Our results indicate that TR-induced subacute toxicity is dose dependent (Figures 1, 2C, Supplementary Figure S5C, Supplementary Tables S4–S6). Besides AST, GLU, and TG, no disturbances in biochemistry and histopathology were observed at the low-dose level, and all observed damages in the high-dose levels were reversible. In addition, the comparison of raw and processed TR indicated that licorice-water extraction with stir-frying processing could effectively reduce TR’s subacute toxicity. Specifically, the serum AST level and liver index in the rat groups treated with processed TR dropped considerably compared with those treated with raw TR (Figure 1B and Supplementary Table S4). The PLT, CK, and LDH levels were normal in rats treated with processed TR and showed biochemical disturbances in rats treated with raw TR (Supplementary Table S5).

The metabolomic profiles of rats treated with TR revealed distinct perturbations in lipid metabolism. The major altered pathways were primary bile acid biosynthesis, steroid biosynthesis, and arachidonic acid metabolism (Figures 2, 3). Serum taurocholic acid level increased after raw TR treatment (Figure 5C), and its accumulation is believed to be highly connected with hepatotoxicity and inflammation (Liu et al., 2014). Interestingly, the relative intensities of 7α-hydroxycholesterol, cholesterol, lathosterol, vitamin D3, calcitriol, and 7α-hydroxy-5 β-cholestan-3-one decreased remarkably after TR administration (Figures 3C, 5C). Together with the remarkably reduced serum TG level (Figure 1B), the results indicated that TR has the potency to be a lipid catabolic agent. Taurocholic acid has been reported as a drug-induced liver injury biomarker in clinical studies (Slopianka et al., 2017; Tian et al., 2021). Differences in hepatotoxic phenotype may correspond to the type of bile acid disruption, and biliary hyperplasia leads to the increase of TCA (Luo et al., 2014). Processed TR could reverse the remarkable disturbance of these biomarkers and alleviate the strong potency induced by raw TR by alleviating perturbations in these pathways (Figures 3C, 5C). The prediction model constructed with the three biomarkers (7α-hydroxycholesterol, calcitriol, and taurocholic acid) by Linear SVM algorithm demonstrated good sensitivity and specificity with an AUC of 0.997, as well as high accuracy in test classification and prediction (Figure 5). The three biomarkers showed clear dose and time dependence, and their levels reverted to normal after 14 days of recovery (Figure 5C). Therefore, the biomarkers demonstrate potential value in TR toxicity prevention, diagnosis, and treatment.

TR administration could lead to lipid metabolism (steroid biosynthesis, primary bile acid biosynthesis, and arachidonic acid pathways) perturbations (Figures 2, 3). The transcriptional regulation of bile acid biosynthesis gene (*Cyp7a1*), biliary excretion gene (*Acbc11*), and arachidonic acid inflammatory activation genes (*cPla2*, *Alox5*, and *Cox2*) confirmed the metabolic alterations (Figure 4). Notably, AhR and its target genes (*Cyp1a1*, *Cyp1a2*, and *Cyp1b1*) were also markedly upregulated after TR administration (Figure 4). AhR is necessary to generate adaptive hepatocyte toxicity (Walisser et al., 2005), and it is the indirect factor of bile acid perturbations (Zhang et al., 2018). AhR activation also induces interleukin biosynthesis in the arachidonic acid metabolic pathway, which leads to the hepatotoxicity of neutrophil infiltration inflammation (Takeda et al., 2017), which is consistent with the liver histopathologic and metabolic findings revealed in our study (Figures 1A, 2, 3). Interestingly, we also observed the nuclear translocation of *Cyp1a1* from the liver samples of the raw TR group, whereas the processed TR group did not indicate AhR activation (Figure 4D). Therefore, AhR could be a key receptor linked to the toxicity phenotype, metabolic regulation, and transcription levels of TR-induced toxicity.

**FIGURE 6:**
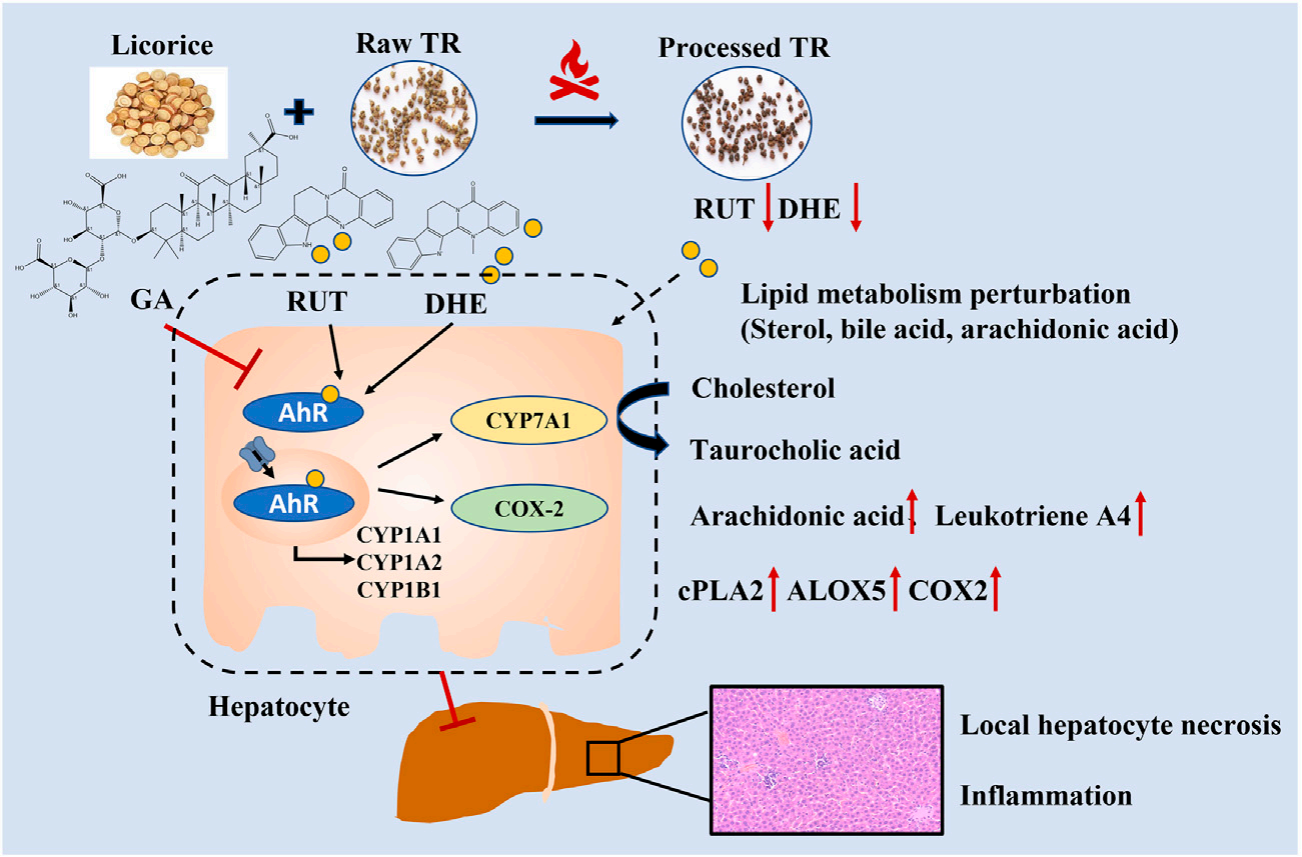
Toxicity and toxicity attenuation mechanisms of raw and processed TR in rats.

RUT and DHE, the main bioactive indoloquinazoline alkaloids of TR, disrupt bile acid homeostasis in an AhR-dependent manner (Zhang et al., 2018). Interestingly, the contents of RUT and DHE decreased remarkably after the processing procedure (Supplementary Table S1), which attenuated AhR activation. The contents of licorice derivates markedly increased in our previous study (Shan Q et al., 2020, Supplementary Table S1). Licorice could detoxify xenobiotics by regulating solubility and enzyme activities in the liver and reducing oxidative stress and anti-inflammatory effects (Al-Dujaili et al., 2011; Kim et al., 2018; Su et al., 2018; Li et al., 2019; Liu et al., 2019). Therefore, the chemical alterations caused by the licorice stir-frying procedure could alleviate TR’s potency on enzyme regulation, alleviate lipid metabolism perturbations in the metabolic level (lipid, bile acid biosynthesis, and inflammation activation), and prevent TR from inducing server toxicity, which explains how stir-frying with licorice aqueous extract could reduce TR’s potential toxicity (Figure 6). Together, the results indicate that TR’s toxicity may attribute to AhR activation with lipid metabolism perturbations, whereas the licorice-processing procedure could alleviate the lipid metabolism perturbations and the agonist action of AhR caused by TR administration.

## Conclusion

*In vivo* toxicology analysis and metabolomics were employed to investigate the toxicity and toxicity attenuation of TR in rats. Although extremely high doses of TR (i.e., 60 fold of the clinically recommended dose) may cause liver toxicity, low-dose TR (i.e., 3 fold of the recommended therapeutic dose) does not cause obvious liver toxicity. Hepatocellular injury was the major toxic phenotype of TR-induced liver damage, indicated as AST and liver index increasing, with local hepatocyte necrosis, focal inflammatory cell infiltration, slight bile duct hyperplasia, and partial hepatocyte vacuolation lesions. Moreover, preprocessing with licorice could effectively reduce TR-induced toxicity and is a valuable step in TR pretreatment before clinical application. Metabolomics profiling revealed that primary bile acid biosynthesis, steroid biosynthesis, and arachidonic acid metabolism were mainly involved in profiling the toxicity metabolic regulatory network; the processing procedure could back-regulate these three pathways, may be in an AHR dependent manner, to alleviate the metabolic perturbations induced by TR. 7α-hydroxycholesterol, calcitriol, and taurocholic acid were screened and validated as the toxic biomarkers of TR for potential clinical translation.

The limitation of our study is that we only conducted hepatic mRNA assay and nuclear translocation by immunofluorescence for the hypothesis of AhR-dependent toxicity and toxicity attenuation mechanism of TR. We assumed that the bioactive components from licorice (i.e., glycyrrhizic acid) could interact with AhR to disrupt the activation caused by RUT and DHE. It would be worth evaluating how much the toxicity of TR is attributed to RUT and DHE, with AhR agonists or inhibitors *in vitro* model, as well as to confirm the correlations between target genes with potential toxic compounds by overexpressing or knockdown *in vitro* or *in vivo*. Compound interactions in cell-viability evaluation would also provide valuable information of the scientific annotation of toxicity attenuation of traditional processing. In addition, clinical trials will be the most convincing support for the biomarkers in further clinical application.

Together, our findings provide insight into TR’s toxicity, potential biomarkers, and processing theory, which can serve as a foundation for TR’s clinical application, as well as TR toxicity prevention, diagnosis, and treatment.

## Data Availability

The raw data supporting the conclusions of this article will be made available by the authors, without undue reservation.

## Abbreviations

AhR, aryl hydrocarbon receptor; ALP, alkaline phosphatase; AST, aspartate aminotransferase; CK, creatine kinase; FC, fold change; FDR, false discovery rate; GA, glycyrrhizic acid; GLU, glucose; H&E, Hematoxylin-eosin; HGB, hemoglobin; HMDB, Human Metabolome Database; KEGG, Kyoto Encyclopedia of Genes and Genomes; LDH, lactate dehydrogenase; PLT, platelet; PCA, principle components analysis; PCR, polymerase chain reaction; PLS-DA, partial least squares discriminant analysis; ROC, receiver operation characteristic; SVM, support vector machine; TG, triglycerides; TR, *Tetradium ruticarpum*; VIP, variable importance in projection.
