# Supplementary material for: Toxicity of Tetradium ruticarpum: Subacute Toxicity Assessment and Metabolomic Identification of Relevant Biomarkers
Source: Front Pharmacol. 2022 Feb 28;13:803855. doi: 10.3389/fphar.2022.803855 (PMC8918793; doi:10.3389/fphar.2022.803855)
Supplement: Supplementary file 1 [file DataSheet1.docx]

**SUPPORTING INFORMATION**

**Toxicity of *Tetradium ruticarpum*: Subacute Toxicity Assessment and Metabolomic Identification of Relevant Biomarkers**

Qiyuan Shan^a,b,1^, Gang Tian^a,1^, Xin Han ^a,1^, Hui Hui^a^, Mai Yamamoto^c^, Min Hao^a^ , Jingwei Wang^d^, Kuilong Wang^a^, Xianan Sang^a^, Luping Qin^a,^*, Guanqun Chen^b,^*, Gang Cao^a,^*

^a^ School of Pharmaceutical Science, Zhejiang Chinese Medical University, Hangzhou, China

^b^ Department of Agricultural, Food and Nutritional Science, University of Alberta, Edmonton, AB, Canada

^c^ Department of Biological Sciences, University of Alberta, Edmonton, AB, Canada

^d^ The Public Platform of Medical Research Center, Academy of Chinese Medical Science, Zhejiang Chinese Medical University, Hangzhou, China

* Correspondence: Luping Qin, lpqin@zcmu.edu.cn; Guanqun Chen, gc24@ualberta.ca; ORCID: 0000-0001-5790-3903; Gang Cao, caogang33@163.com

^1^ These authors equally contributed to the current study.

**Abbreviations**

ALP, Alkaline phosphatase; AST, Aspartate aminotransferase; CK, Creatine kinase; GA, Glycyrrhizic acid; GLU, Glucose; H&E, Hematoxylin-eosin; HGB, Hemoglobin; LDH, Lactate dehydrogenase; PLT, platelet; RT-qPCR, quantitative Real-time Polymerase chain reaction; RBC, red blood cell; TG, triglycerides; TR, *Tetradium ruticarpum*; WBC, White blood cell.

**Supplementary Figures**


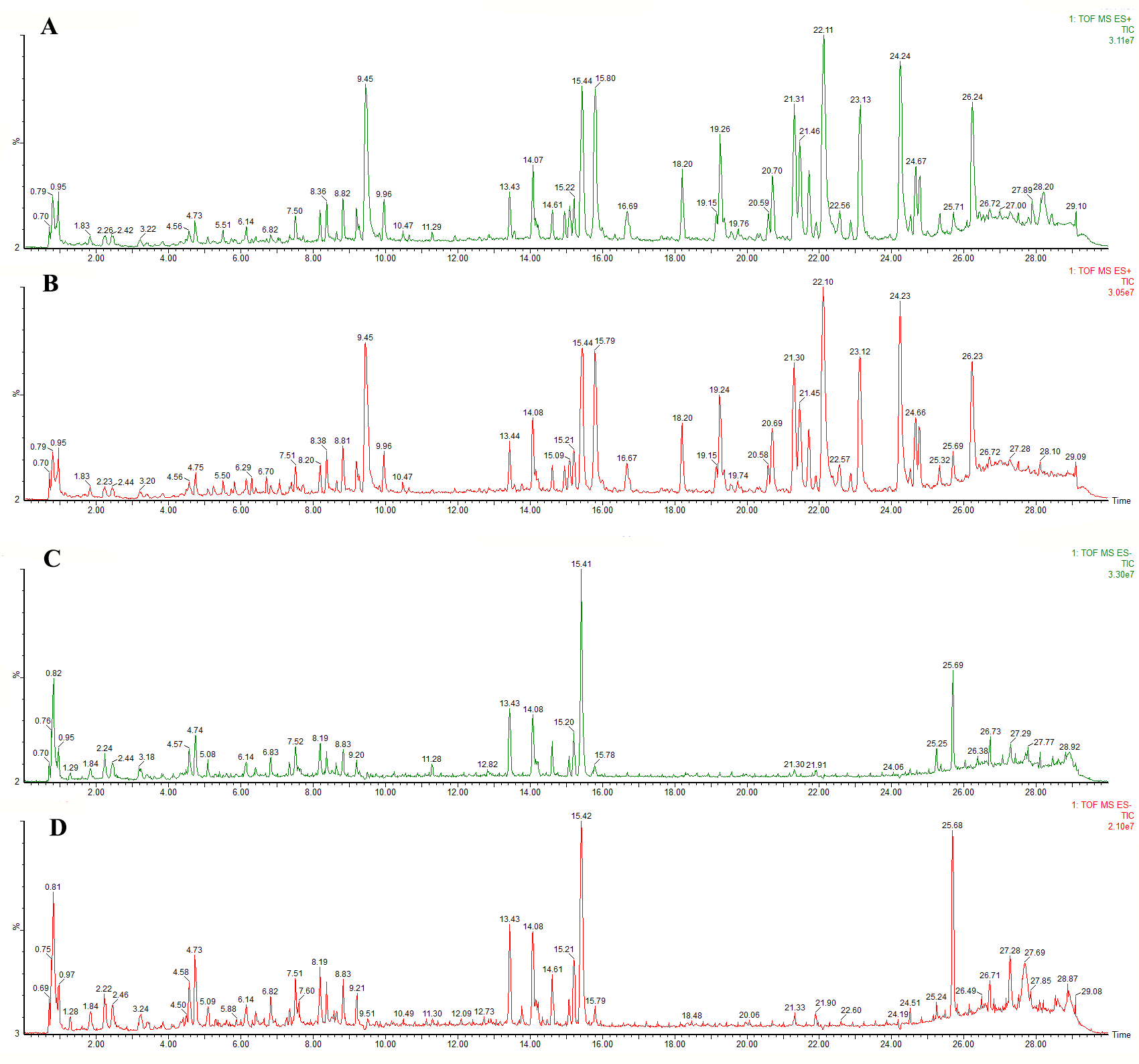


**Figure S1.** The total ion chromatograph of raw and processed *Tetradium ruticarpum* (TR) extracts in positive ion mode (A: raw TR, B: processed TR) and negative ion mode (C: raw TR and D: processed TR).


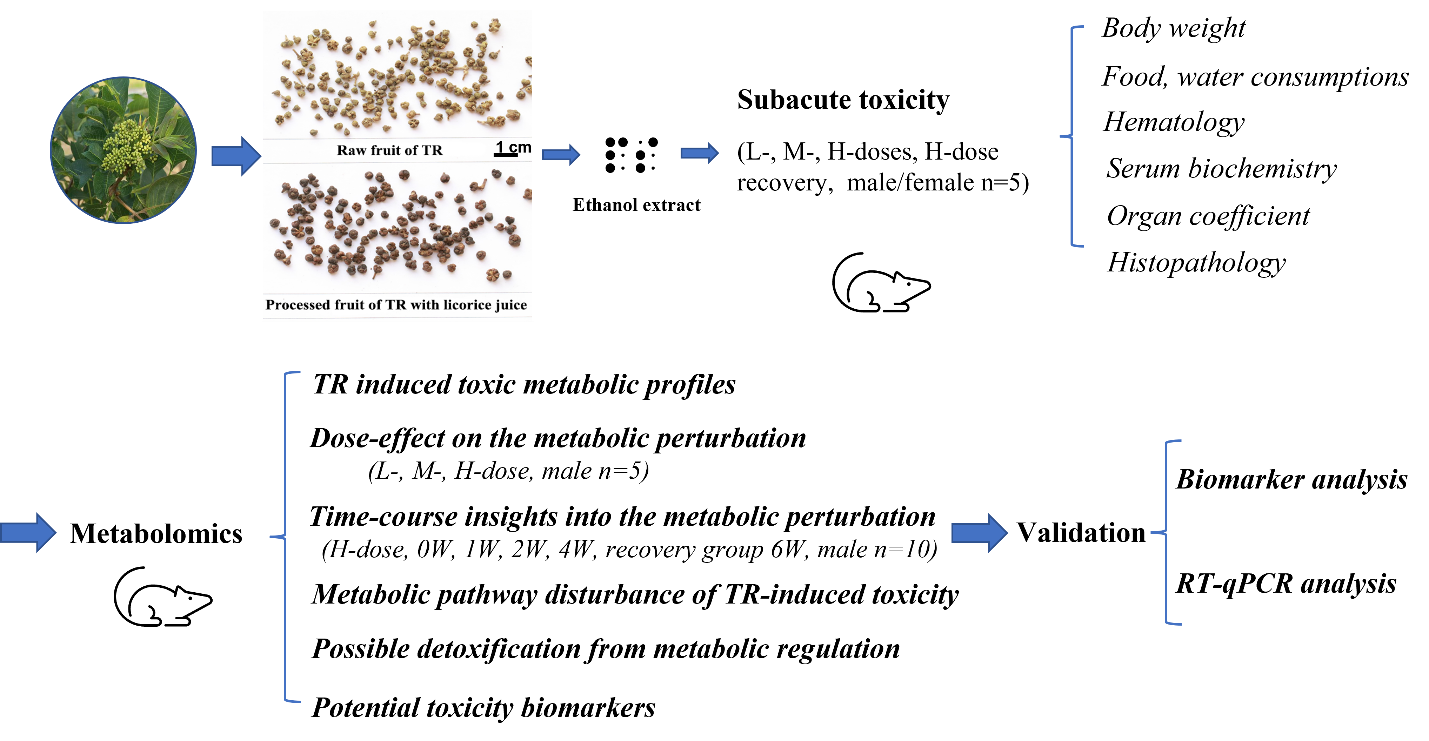


**Figure S2**. Experimental design of subacute toxicity and metabolomics study of TR administration.


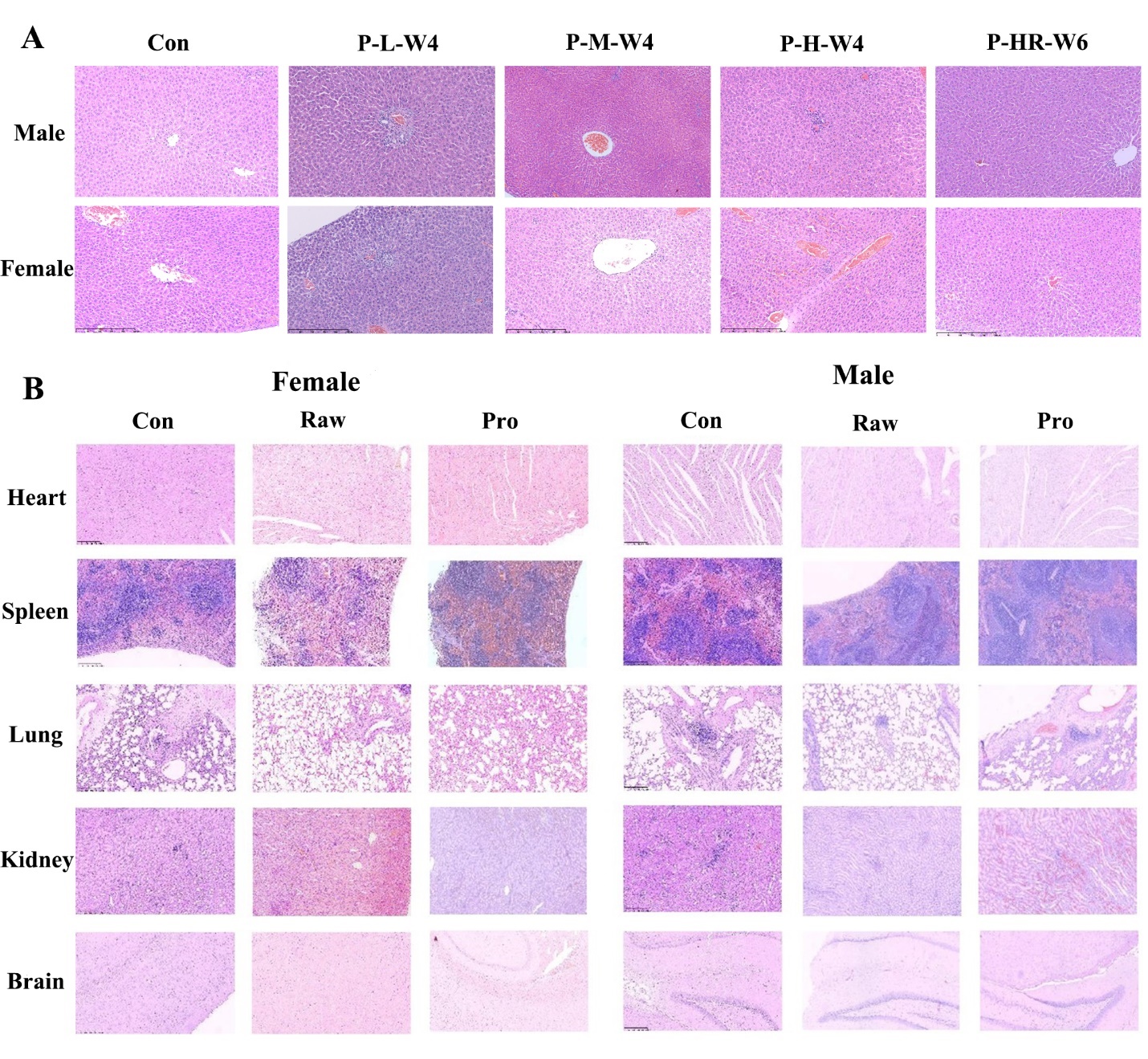


**Figure S3**. Histopathological results of TR treatment. **(A)** Liver histopathological results of female and male rats treated with processed (P) TR for 28 days (Con: control, L: low dose, M: medium dose, H: high dose), recovery groups (HR: 28 days of high dose administration and followed by 14-day recovery), and control groups were administrated with normal saline (×100). **(B)** Other main organ histopathological results of female and male rats treated with raw and processed TR for 28 days (×100).


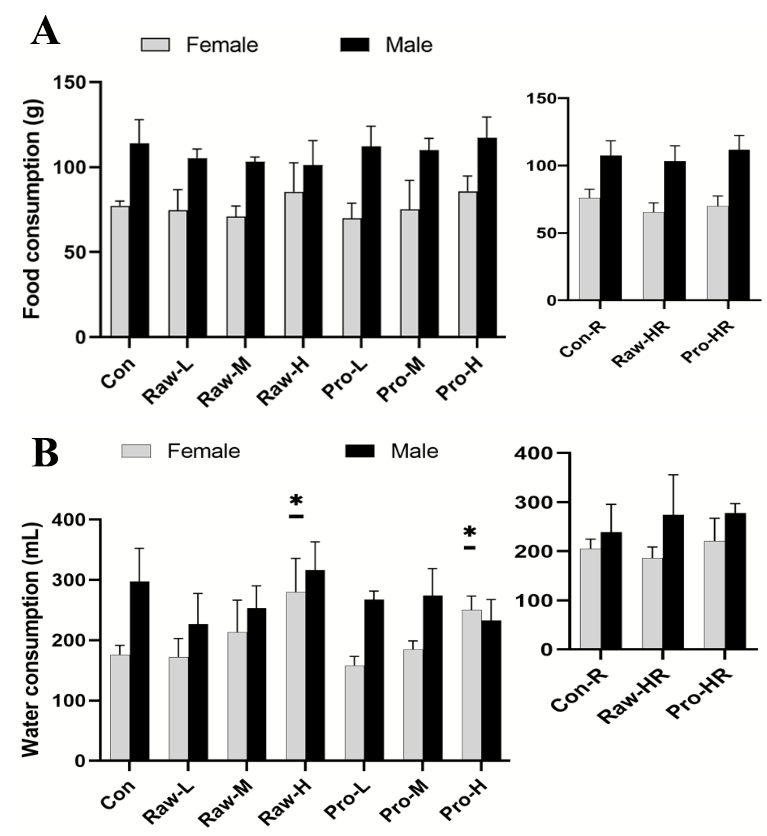


**Figure S4**. Food and water consumptions of rats orally treated with 70% ethanol extract of *Tetradium ruticarpum* (TR) for 28 days and 14-day recovery. Raw: raw TR, Pro: processed TR, with L: low dose (0.15 g/kg), M: moderate dose (1.5 g/kg), and H: high dose (3.0 g/kg); Con-R: control for 42 days, Raw-HR/Pro-HR: treated by high dose of raw/processed TR (3.0 g/kg) for 28 days and followed by 14 days recovery.


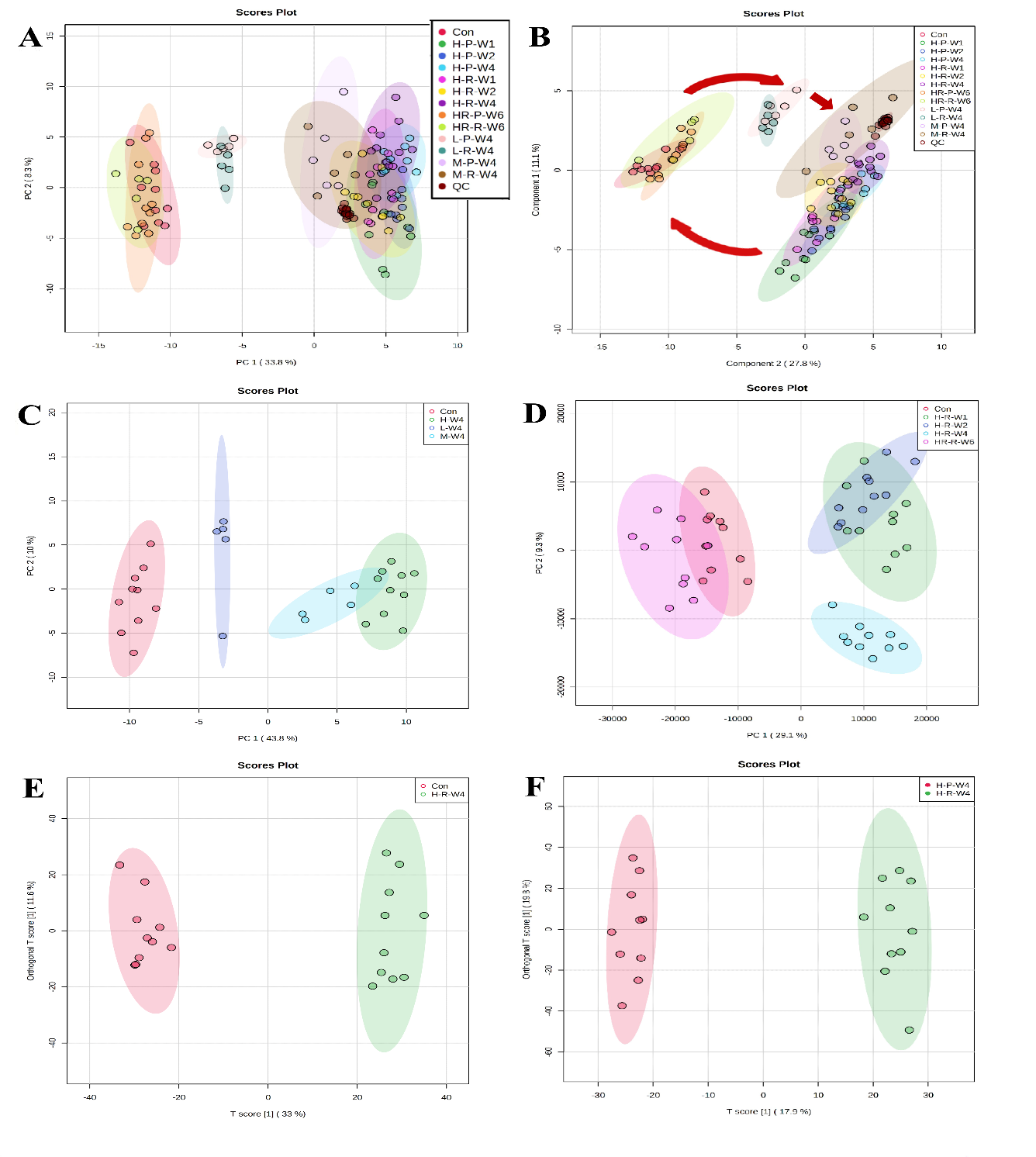


**Figure S5.** The clustering profile of rat serum samples after TR treatment in the negative ion mode. **(A)** Principle component analysis (PCA) score plot of the whole treating groups for different time course including raw (R) and processed (P) TR groups, and control (Con) group as well as quality control (QC) samples; **(B)** the clustering results of rat serum samples after raw and processed TR treatment by sparse Partial Least Squares Discriminant Analysis (sPLS-DA) method; **(C)** the dose-effect groups clustering results of TR raw samples **(D)** the time-course groups clustering results of TR raw samples; **(E)** the control group vs. high dose of raw TR treatment group for 28 days; **(F)** the raw group vs. processed group of TR treatment for 28 days.

**Supplementary Tables**

**Table S1.** Determination of the main bioactive components of raw and processed TR (mg/g, n=3)

|  | Dehydroevodiamine | Limonin | Evodiamine | Rutaecarpine | Evocarpine | Liquiritin | Glycyrrhizic acid | 18β-Glycyrrhetinic acid |
| --- | --- | --- | --- | --- | --- | --- | --- | --- |
| Raw TR | 4.39 ± 0.42 | 7.71 ± 0.09 | 15.67 ± 1.14 | 8.86 ± 0.08 | 2.28 ± 0.18 | nd | nd | nd |
| Processed TR | 3.56 ± 0.09 | 7.02 ± 0.42 | 14.27 ± 0.94 | 7.71 ± 0.03 | 2.02 ± 0.08 | 0.14 ± 0.02 | 0.62 ± 0.02 | 0.014 ± 0.00 |

**Table S2.** The primer sequences design of targeted genes.

| Gene | Genbank Accession | Primer Sequences(5'to3') | length |
| --- | --- | --- | --- |
| Ahr | NM_001308254 | cagtgtagagcacaagtcagag | 127 |
|  |  | ggatagtggaagacgcatagaag |  |
| Cyp1a1 | NM_012540 | gggcaagcgaaagtgcattggg | 186 |
|  |  | tgctgaggaccagaagaccgca |  |
| Cyp1a2 | NM_012541 | cacagcacaacgagggacacct | 215 |
|  |  | atgcaccggcgctttcccaa |  |
| Cyp1b1 | NM_012940 | agcgctgtatgcttcggctgt | 151 |
|  |  | tgcgcaccgggttgggaaaa |  |
| Cyp7a1 | NM_012942 | tctttccggcactggtggca | 196 |
|  |  | aggtgcgtcttggccttctcca |  |
| Sc5d | NM_053642 | ataggggcctgcaccacagact | 217 |
|  |  | accaccttgtgcagcgggaa |  |
| Abcb11 | NM_031760 | tggacggccatgacattcgc | 194 |
|  |  | ttgctgcggcagggccataa |  |
| cPLA2 | NM_133551 | aactcgacgcagcggtagca | 195 |
|  |  | ggctggtgtcacttggcctt |  |
| Alox5 | NM_012822 | agaccgtggccgctcatgtt | 188 |
|  |  | ttgcgctcggcaatcacgct |  |
| Cox-2 | NM_017232 | gcgctcagccatgcagcaaa | 171 |
|  |  | ttggggtgggcttcagcagt |  |
| β-actin | B4002409.2 | caggaaggaaggctggaag | 55 |
|  |  | cgggaaatcgtgcgtgac |  |

**Table S3**. The body weight of rats by oral administration of the ethanol extract of *Tetradium ruticarpum* (TR) for 28 days and 14 days recovery stage.

| **Body weight(g)** | Control | Raw TR (g/kg) | | | Processed TR (g/kg) | | | Control-R | Raw TR-R (g/kg) | Processed TR-R (g/kg) |
| --- | --- | --- | --- | --- | --- | --- | --- | --- | --- | --- |
|  | 0 | 1.5 | 15 | 30 | 1.5 | 15 | 30 | 0 | 30 with recovery | 30 with recovery |
| **Male** |  |  |  |  |  |  |  |  |  |  |
| Day 1 | 262.20 ± 5.12 | 265.80 ± 11.30 | 267.80 ± 10.18 | 268.60 ± 13.32 | 273.80 ± 8.90 | 271.20 ± 10.94 | 272.60 ± 11.63 | 268.20 ± 6.53 | 265.40 ± 15.08 | 261.60 ± 13.70 |
| Day 7 | 313.80 ± 5.36 | 302.80 ± 9.63 | 307.20 ± 12.03 | 306.40 ± 20.56 | 316.00 ± 5.61 | 309.80 ± 6.50 | 320.80 ± 11.12 | 306.80 ± 14.58 | 299.40 ± 19.50 | 302.80 ± 7.26 |
| Day 14 | 358.80 ± 9.78 | 340.80 ± 11.41 | 342.80 ± 11.19 | 343.60 ± 32.88 | 363.40 ± 7.80 | 364.20 ± 7.82 | 372.80 ± 10.03 | 343.40 ± 22.50 | 333.80 ± 21.34 | 341.40 ± 7.57 |
| Day 21 | 394.20 ± 13.35 | 367.80 ± 11.03 | 366.80 ± 20.17 | 352.60 ± 47.55^*^ | 398.60 ± 7.30 | 384.40 ± 9.40 | 397.20 ± 7.33 | 374.20 ± 30.85 | 363.80 ± 30.29 | 363.20 ± 5.89 |
| Day 28 | 401.60 ± 13.15 | 372.00 ± 7.31 | 366.60 ± 19.13 | 355.60 ± 41.14^**^ | 406.60 ± 7.57 | 385.80 ± 6.26 | 389.00 ± 7.58 | 401.60 ± 34.20 | 393.80 ± 21.68 | 393.80 ± 12.30 |
| Day 35 | - | - | - | - | - | - | - | 418.20 ± 28.53 | 407.60 ± 32.31 | 412.00 ± 15.41 |
| Day 42 | - | - | - | - | - | - | - | 438.40 ± 26.80 | 424.60 ± 28.05 | 438.60 ± 20.42 |
| **Female** |  |  |  |  |  |  |  |  |  |  |
| Day 1 | 206.40 ± 9.32 | 206.60 ± 10.64 | 202.00 ± 10.27 | 204.60 ± 15.18 | 208.00 ± 7.91 | 203.20 ± 3.35 | 209.80 ± 15.51 | 201.60 ± 9.07 | 206.20 ± 8.35 | 198.60 ± 10.45 |
| Day 7 | 221.20 ± 8.98 | 221.20 ± 15.35 | 214.20 ± 20.32 | 220.20 ± 6.94 | 224.20 ± 14.58 | 224.20 ± 6.50 | 224.80 ± 9.09 | 221.60 ± 11.61 | 225.60 ± 8.20 | 215.20 ± 12.15 |
| Day 14 | 241.40 ± 13.70 | 241.20 ± 15.96 | 234.60 ± 18.74 | 235.80 ± 11.65 | 227.40 ± 10.64 | 233.20 ± 7.92 | 239.60 ± 11.15 | 236.00 ± 14.71 | 237.80 ± 8.47 | 231.80 ± 10.28 |
| Day 21 | 255.60 ± 8.41 | 257.40 ± 19.82 | 246.00 ± 21.77 | 243.20 ± 12.46 | 246.60 ± 13.24 | 246.40 ± 5.22 | 245.20 ± 12.21 | 252.60 ± 9.29 | 253.80 ± 14.96 | 239.60 ± 9.26 |
| Day 28 | 250.60 ± 8.85 | 254.20 ± 20.40 | 243.20 ± 25.11 | 239.00 ± 11.25 | 241.20 ± 12.87 | 244.00 ± 7.68 | 242.60 ± 8.62 | 260.60 ± 13.56 | 262.80 ± 19.01 | 250.40 ± 5.73 |
| Day 35 | - | - | - | - | - | - | - | 278.80 ± 16.81 | 269.60 ± 18.80 | 252.20 ± 9.93^*^ |
| Day 42 | - | - | - | - | - | - | - | 280.80 ± 21.09 | 277.60 ± 24.34 | 257.40 ± 13.28 |

All data are expressed as means ± standard deviation (female/male n = 5). ^*^ *p* < 0.05, ^**^ *p* < 0.01, ^***^ *p* < 0.001, when compared with the relative control groups, or the control-R group (for the recovery groups).

**Table S4**. The effect of the 70% ethanol extract of TR on organ weights of rats after 28-day administration and followed by a 14-day recovery stage.

| **Organ/weight ratio (%)** | Control | Raw TR (g/kg) | | | Processed TR (g/kg) | | | Control-R | Raw TR-R (g/kg) | Processed TR-R (g/kg) |
| --- | --- | --- | --- | --- | --- | --- | --- | --- | --- | --- |
|  | 0 | 1.5 | 15 | 30 | 1.5 | 15 | 30 | 0 | 30 with recovery | 30 with recovery |
| **Male** |  |  |  |  |  |  |  |  |  |  |
| heart | 0.28 ± 0.02 | 0.30 ± 0.02 | 0.29 ± 0.03 | 0.29 ± 0.02 | 0.29 ± 0.03 | 0.30 ± 0.02 | 0.27 ± 0.02 | 0.30 ± 0.02 | 0.28 ± 0.03 | 0.97 ± 1.50 |
| liver | 3.03 ± 0.25 | 2.93 ± 0.17 | 3.20 ± 0.35 | 3.99±0.08*** | 2.86 ± 0.09 | 3.24 ± 0.14 | 3.66 ± 0.15***## | 2.97 ± 0.38 | 2.74 ± 0.09 | 3.12 ± 0.20 |
| spleen | 0.24 ± 0.05 | 0.27 ± 0.02 | 0.25 ± 0.02 | 0.22 ± 0.04 | 0.26 ± 0.06 | 0.22 ± 0.03 | 0.22 ± 0.05 | 0.26 ± 0.03 | 0.25 ± 0.03 | 0.24 ± 0.02 |
| lung | 0.38 ± 0.04 | 0.44 ± 0.05 | 0.42 ± 0.08 | 0.43 ± 0.04 | 0.41 ± 0.05 | 0.40 ± 0.04 | 0.38 ± 0.04 | 0.40 ± 0.05 | 0.40 ± 0.03 | 0.42 ± 0.06 |
| kidney | 0.66 ± 0.01 | 0.69 ± 0.05 | 0.68 ± 0.11 | 0.79±0.04* | 0.66 ± 0.04 | 0.65 ± 0.06 | 0.68 ± 0.03 | 0.66 ± 0.06 | 0.62 ± 0.07 | 0.70 ± 0.08 |
| brain | 0.49 ± 0.03 | 0.53 ± 0.02 | 0.55 ± 0.04 | 0.58 ± 0.04 | 0.51 ± 0.01 | 0.52 ± 0.02 | 0.50 ± 0.02 | 0.51 ± 0.03 | 0.48 ± 0.04 | 0.49 ± 0.11 |
| testicle | 0.88 ± 0.04 | 0.94 ± 0.08 | 0.97 ± 0.04 | 1.03 ± 0.12* | 0.92 ± 0.05 | 0.92 ± 0.10 | 0.96 ± 0.09 | 0.94 ± 0.02 | 0.84 ± 0.04 | 0.96 ± 0.09 |
| epididymis | 0.25 ± 0.02 | 0.29 ± 0.03 | 0.31 ± 0.03** | 0.32 ± 0.03** | 0.27 ± 0.03 | 0.28 ± 0.02 | 0.29 ± 0.02 | 0.36 ± 0.03 | 0.30 ± 0.03* | 0.34 ± 0.02 |
| **Female** |  |  |  |  |  |  |  |  |  |  |
| heart | 0.29 ± 0.02 | 0.31 ± 0.02 | 0.30 ± 0.03 | 0.31 ± 0.03 | 0.32 ± 0.05 | 0.30 ± 0.01 | 0.29 ± 0.02 | 0.31 ± 0.03 | 0.33 ± 0.04 | 0.30 ± 0.02 |
| liver | 3.04 ± 0.21 | 2.97 ± 0.16 | 3.43 ± 0.27* | 4.31±0.10*** | 3.06 ± 0.31 | 3.26 ± 0.15 | 3.95 ± 0.27***# | 2.94 ± 0.14 | 3.08 ± 0.38 | 3.05 ± 0.26 |
| spleen | 0.26 ± 0.03 | 0.30 ± 0.04 | 0.27 ± 0.04 | 0.28 ± 0.05 | 0.28 ± 0.04 | 0.28 ± 0.06 | 0.23 ± 0.03 | 0.25 ± 0.05 | 0.24 ± 0.06 | 0.24 ± 0.04 |
| lung | 0.48 ± 0.03 | 0.54 ± 0.05 | 0.53 ± 0.06 | 0.53 ± 0.07 | 0.54 ± 0.07 | 0.46 ± 0.07 | 0.47 ± 0.06 | 0.48 ± 0.03 | 0.51 ± 0.11 | 0.49 ± 0.03 |
| kidney | 0.67 ± 0.04 | 0.66 ± 0.06 | 0.67 ± 0.04 | 0.77 ± 0.11 | 0.68 ± 0.05 | 0.68 ± 0.06 | 0.70 ± 0.03 | 0.62 ± 0.05 | 0.67 ± 0.07 | 0.64 ± 0.03 |
| brain | 0.72 ± 0.06 | 0.76 ± 0.04 | 0.79 ± 0.06 | 0.76 ± 0.06 | 0.76 ± 0.05 | 0.76 ± 0.05 | 0.77 ± 0.05 | 0.70 ± 0.05 | 0.73 ± 0.11 | 0.74 ± 0.02 |
| uterus | 0.27 ± 0.09 | 0.27 ± 0.14 | 0.24 ± 0.06 | 0.21 ± 0.05 | 0.23 ± 0.08 | 0.26 ± 0.13 | 0.27 ± 0.08 | 0.24 ± 0.09 | 0.24 ± 0.08 | 0.22 ± 0.04 |
| ovary | 0.08 ± 0.01 | 0.07 ± 0.01 | 0.08 ± 0.01 | 0.07 ± 0.01 | 0.09 ± 0.01 | 0.07 ± 0.02 | 0.07 ± 0.01 | 0.08 ± 0.02 | 0.08 ± 0.02 | 0.07 ± 0.01 |

All data are expressed as means ± standard deviation (female/male n = 5). ^*^ *p* < 0.05, ^**^ *p* < 0.01, ^***^ *p* < 0.001, when compared with the relative control groups. ^#^ *p* < 0.05, ^##^ *p* < 0.01, when compared with groups treated with raw TR at the same dose.

**Table S5**. Hematological parameters of rats administrated with raw and processed TR for 28-day subacute toxicity study and followed by a 14-day recovery stage.

| **TR dose (g/kg)** | Control | Raw TR | | | | Processed TR | | | |
| --- | --- | --- | --- | --- | --- | --- | --- | --- | --- |
|  | 0 | 1.5 | 15 | 30 | 30 with recovery | 1.5 | 15 | 30 | 30 with recovery |
| **Male** |  |  |  |  |  |  |  |  |  |
| WBC(10^9/L) | 4.67±0.69 | 5.57±1.13 | 6.39±0.98 | 4.78±2.17 | 5.47±2.04 | 4.62±0.83 | 5.32±1.24 | 4.35±2.00 | 5.65±1.73 |
| NEUT(%) | 11.60±2.14 | 14.84±4.08 | 19.50±11.29 | 16.32±9.25 | 14.28±0.52 | 20.42±7.94 | 14.42±2.48 | 13.52±2.96 | 13.38±3.64 |
| LYMPH(%) | 84.00±1.90 | 82.02±4.40 | 76.46±11.79 | 79.88±11.48 | 81.54±1.11 | 75.04±8.54 | 81.66±2.71 | 81.88±2.88 | 82.92±4.11 |
| MONO(%) | 1.60±0.23 | 1.72±0.58 | 2.30±0.71 | 2.50±2.14 | 1.82±0.22 | 2.68±0.78 | 2.10±0.23 | 2.80±1.66 | 1.46±0.22 |
| EOS(%) | 1.98±0.81 | 0.88±0.33 | 0.92±0.30 | 0.66±0.25 | 1.74±0.66 | 1.28±0.36 | 1.18±0.27 | 0.84±0.41 | 1.60±0.57 |
| BASO(%) | 0.10±0.07 | 0.14±0.05 | 0.08±0.04 | 0.10±0.07 | 0.10±0.07 | 0.08±0.04 | 0.10±0.07 | 0.06±0.05 | 0.08±0.04 |
| LUC(%) | 0.78±0.28 | 0.44±0.05 | 0.72±0.36 | 0.54±0.25 | 0.50±0.20 | 0.54±0.26 | 0.52±0.08 | 0.86±0.21 | 0.48±0.24 |
| RBC (10^12/L) | 7.90±0.24 | 8.10±0.29 | 7.98±0.34 | 8.26±0.43 | 7.84±0.27 | 7.60±0.48 | 8.07±0.21 | 8.12±0.25 | 8.24±0.56 |
| HGB(g/L) | 142.60±4.88 | 143.20±3.35 | 142.20±3.27 | 145.20±9.20 | 135.40±5.18 | 137.80±3.96 | 144.20±3.03 | 146.80±1.92 | 138.80±7.19 |
| HCT(%) | 45.60±1.38 | 47.66±1.66 | 46.74±1.28 | 47.74±2.34 | 45.20±1.25 | 45.18±1.63 | 47.56±1.20 | 47.58±0.77 | 46.02±2.55 |
| MCV(fL) | 57.72±1.53 | 58.82±1.47 | 58.54±0.98 | 57.82±1.35 | 57.66±1.86 | 59.58±2.03 | 58.90±0.95 | 58.66±0.99 | 55.92±1.95 |
| MCH(Pg) | 18.06±0.44 | 17.70±0.42 | 17.86±0.52 | 17.56±0.49 | 17.30±0.80 | 18.18±0.81 | 17.90±0.16 | 18.10±0.54 | 16.90±0.96* |
| MCHC(g/L) | 312.80±4.09 | 300.80±5.93* | 304.80±5.26 | 303.60±5.03* | 299.80±7.09* | 305.00±4.36 | 303.60±3.44 | 308.40±5.77 | 302.00±7.65* |
| PLT(10^9/L) | 1063.80±40.52 | 1061.40±64.62 | 1056.20±77.31 | 1441.40±188.29*** | 1022.20±118.77 | 928.80±42.65 | 1046.00±107.08 | 1123.60±147.94 | 1009.40±82.55 |
| PCT(%) | 0.85±0.03 | 1.07±0.04* | 1.00±0.10 | 1.34±0.20*** | 1.00±0.16 | 0.86±0.03 | 0.89±0.09 | 0.78±0.10 | 1.03±0.10 |
| PDW(%) | 49.20±4.08 | 49.98±1.33 | 50.50±1.39 | 50.28±3.35 | 45.78±6.84 | 54.50±2.37 | 53.52±1.82 | 44.50±3.96 | 51.40±5.66 |
| **Female** |  |  |  |  |  |  |  |  |  |
| WBC(10^9/L) | 2.84±0.78 | 3.38±0.66 | 2.46±0.95 | 3.32±1.51 | 3.22±1.12 | 3.95±2.39 | 4.01±1.48 | 3.04±0.36 | 3.11±0.93 |
| NEUT(%) | 15.10±7.27 | 13.18±1.77 | 17.74±8.34 | 16.16±10.00 | 14.60±1.58 | 12.14±5.72 | 14.26±2.97 | 8.94±2.31 | 8.78±1.84 |
| LYMPH(%) | 80.26±7.77 | 81.86±2.74 | 78.90±8.00 | 79.94±11.63 | 81.60±2.29 | 83.68±6.70 | 81.28±3.62 | 87.80±2.78 | 87.80±1.64 |
| MONO(%) | 1.66±0.38 | 1.88±0.34 | 1.50±0.38 | 2.12±0.75 | 1.83±0.73 | 1.92±0.73 | 2.36±0.84 | 1.42±0.48 | 1.30±0.49 |
| EOS(%) | 2.30±0.53 | 2.36±0.39 | 0.98±0.58* | 1.02±1.12* | 1.38±0.79 | 1.68±0.96 | 1.00±0.70* | 0.98±0.34* | 1.32±0.58 |
| BASO(%) | 0.10±0.07 | 0.14±0.05 | 0.18±0.08 | 0.12±0.08 | 0.08±0.04 | 0.12±0.08 | 0.18±0.08 | 0.14±0.11 | 0.10±0.07 |
| LUC(%) | 0.58±0.18 | 0.60±0.26 | 0.72±0.33 | 0.60±0.32 | 0.50±0.32 | 0.48±0.27 | 0.96±0.32 | 0.70±0.12 | 0.70±0.25 |
| RBC (10^12/L) | 7.59±0.23 | 7.46±0.39 | 7.34±0.32 | 6.95±0.62 | 7.63±0.84 | 7.34±0.21 | 7.43±0.21 | 7.74±0.38 | 7.53±0.19 |
| HGB(g/L) | 136.60±2.51 | 131.60±6.27 | 130.60±5.68 | 122.00±12.69* | 133.25±13.99 | 130.80±4.32 | 133.00±3.81 | 139.00±6.96 | 131.80±4.92 |
| HCT(%) | 43.36±0.66 | 44.04±2.13 | 42.74±1.81 | 40.72±3.56 | 42.90±4.85 | 43.06±0.64 | 43.02±0.91 | 45.60±2.63 | 42.64±1.74 |
| MCV(fL) | 57.20±1.44 | 59.10±1.24 | 58.28±1.20 | 58.64±1.27 | 56.25±0.52 | 58.68±1.29 | 57.96±1.28 | 58.90±2.18 | 56.58±1.02 |
| MCH(Pg) | 18.08±0.75 | 17.64±0.36 | 17.78±0.50 | 17.54±0.55 | 17.50±0.19 | 17.84±0.40 | 17.90±0.28 | 17.98±0.64 | 17.48±0.26 |
| MCHC(g/L) | 315.40±6.23 | 298.20±3.11*** | 305.40±4.04* | 299.60±7.27*** | 311.25±4.32 | 303.80±6.53* | 309.20±4.66 | 305.20±5.76* | 309.20±4.66 |
| PLT(10^9/L) | 998.20±105.00 | 1082.00±69.86 | 1171.40±142.21 | 1553.20±207.26*** | 1126.75±134.43 | 1115.80±71.30 | 1178.00±148.78 | 1190.40±72.86 | 1091.40±77.44 |
| PCT(%) | 0.84±0.08 | 1.13±0.07* | 1.17±0.15** | 1.49±0.21*** | 1.13±0.18* | 1.06±0.08 | 1.12±0.15* | 1.11±0.08* | 1.16±0.20** |
| PDW(%) | 47.02±5.15 | 41.50±1.19 | 44.02±2.81 | 45.04±2.15 | 44.08±1.21 | 46.78±2.19 | 48.00±2.01 | 46.28±1.68 | 44.58±2.04 |

All data are expressed as means ± standard deviation (n = 5). ^*^ *p* < 0.05, ^**^ *p* < 0.01, ^***^ *p* < 0.001, when compared with the control group.

**Table S6**. Serum biochemistry in rats administrated with raw and processed TR for 28-day subacute toxicity study and followed by a 14-day recovery stage.

| **TR dose (g/kg)** | Control | Raw TR | | | | Processed TR | | | |
| --- | --- | --- | --- | --- | --- | --- | --- | --- | --- |
|  | 0 | 1.5 | 15 | 30 | 30 with recovery | 1.5 | 15 | 30 | 30 with recovery |
| **Male** |  |  |  |  |  |  |  |  |  |
| ALT (U/L) | 30.50±2.75 | 21.34±3.08* | 18.46±4.17* | 19.74±3.44* | 30.24±4.77 | 23.38±2.99 | 22.04±5.64 | 19.04±5.01 | 37.24±12.78 |
| AST (U/L) | 76.32±7.36 | 134.42±19.70* | 128.08±31.74* | 162.16±35.01*** | 101.56±17.33 | 132.74±29.61* | 136.40±48.33** | 119.64±18.73# | 95.98±12.88 |
| TBIL (μmol/L) | 1.32±0.20 | 1.48±0.25 | 1.24±0.17 | 1.52±0.22 | 1.48±0.29 | 1.48±0.26 | 1.30±0.24 | 1.60±0.25 | 1.40±0.22 |
| ALP (U/L) | 170.04±14.87 | 128.48±6.40 | 122.52±20.83* | 105.34±15.33** | 118.24±30.93* | 145.94±33.11 | 124.68±37.29* | 122.82±16.89** | 116.02±33.85** |
| TBA (μmol/L) | 21.34±9.50 | 13.02±14.48 | 23.00±14.48 | 10.02±9.69 | 13.04±6.86 | 12.22±3.98 | 16.48±10.02 | 22.70±16.21 | 15.82±4.93 |
| GLU (mmol/L) | 6.32±0.20 | 3.83±0.52** | 4.25±0.35*** | 4.21±0.22*** | 5.80±0.30 | 4.69±0.80 | 4.74±0.17*** | 4.40±0.27** | 5.89±0.52 |
| BUN (mmol/L) | 5.27±0.15 | 5.90±1.29 | 6.19±0.93 | 6.24±0.91 | 6.08±1.20 | 5.56±0.64 | 5.50±1.37 | 6.58±0.80 | 6.41±0.64 |
| CREA (μmol/L) | 27.64±1.98 | 28.28±3.16 | 26.60±2.87 | 27.16±2.20 | 32.08±6.06 | 26.86±1.08 | 26.32±2.37 | 28.16±1.09 | 29.68±3.57 |
| CHOl (mg/dl) | 1.65±0.33 | 1.45±0.30 | 1.40±0.17 | 1.56±0.45 | 1.49±0.27 | 1.70±0.17 | 1.35±0.12 | 1.49±0.25 | 1.54±0.36 |
| TG (mmol/L) | 0.70±0.15 | 0.37±0.04 | 0.34±0.05 | 0.28±0.06* | 0.76±0.16 | 0.50±0.24 | 0.52±0.18 | 0.29±0.08* | 0.59±0.20 |
| CK (U/L) | 310.36±59.38 | 738.64±87.51** | 768.12±307.85 | 1035.16±284.64 | 588.18±206.32 | 788.14±231.81 | 716.24±279.38 | 594.54±125.25^#^ | 472.32±126.72 |
| LDH (U/L) | 474.40±175.23 | 1877.26±218.44*** | 1,802.12±785.85 | 2,370.06±748.79 | 871.60±315.05 | 1,702.58±565.68 | 1,612.72±693.41 | 1,356.28±320.04^#^ | 614.72±258.84 |
| **Female** |  |  |  |  |  |  |  |  |  |
| ALT (U/L) | 21.88±8.91 | 19.16±2.25 | 18.82±2.73 | 23.20±7.19 | 24.04±2.69 | 23.04±4.89 | 23.46±6.22 | 16.86±3.74 | 25.44±5.89 |
| AST (U/L) | 88.68±18.44 | 97.22±14.20 | 88.72±9.50 | 132.38±34.79 | 87.64±19.25 | 94.72±10.87 | 121.74±31.28 | 92.40±20.93 | 90.10±6.47 |
| TBIL (μmol/L) | 1.04±0.23 | 1.04±0.23 | 1.68±0.27* | 1.70±0.73 | 1.34±0.15 | 1.42±0.66 | 1.60±0.41 | 1.70±0.25* | 1.04±0.11 |
| ALP (U/L) | 71.72±14.17 | 73.86±16.82 | 56.36±11.81 | 68.42±33.10 | 63.56±15.92 | 65.30±10.74 | 76.46±9.45 | 64.72±32.15 | 53.80±13.14 |
| TBA (μmol/L) | 7.88±4.91 | 6.56±2.48 | 12.80±7.43 | 9.94±3.20 | 17.78±9.21 | 14.08±10.35 | 45.24±49.04 | 26.38±19.47 | 16.44±11.94 |
| GLU (mmol/L) | 5.24±1.20 | 4.63±0.22 | 4.65±0.53 | 5.41±0.73 | 6.73±0.33 | 5.12±0.43 | 5.35±0.23 | 4.31±0.74 | 6.35±0.47 |
| BUN (mmol/L) | 6.10±1.09 | 6.51±1.17 | 5.98±0.85 | 7.22±0.83 | 8.00±1.47 | 6.20±1.93 | 6.45±0.67 | 5.67±0.69 | 8.77±3.05 |
| CREA (μmol/L) | 30.54±3.49 | 30.42±5.75 | 30.46±1.13 | 30.70±2.02 | 44.28±13.52 | 34.80±8.67 | 32.76±2.19 | 29.44±4.89 | 38.98±10.90 |
| CHOl (mg/dl) | 1.55±0.41 | 1.48±0.21 | 1.51±0.31 | 2.09±0.44* | 1.72±0.24 | 1.57±0.46 | 1.47±0.20 | 2.00±0.37 | 1.87±0.38 |
| TG (mmol/L) | 0.53±0.14 | 0.39±0.07 | 0.32±0.04* | 0.40±0.08 | 0.52±0.16 | 0.36±0.05* | 0.38±0.03 | 0.33±0.08* | 0.54±0.09 |
| CK (U/L) | 379.18±153.84 | 439.00±141.04 | 313.50±8.56 | 384.82±109.63 | 249.36±117.25 | 308.34±51.20 | 542.76±274.46 | 449.40±198.15 | 364.72±98.22 |
| LDH (U/L) | 744.58±408.56 | 853.94±317.35 | 653.46±81.31 | 1,032.58±531.90 | 304.94±182.72 | 624.40±150.44 | 1,146.12±665.58 | 915.26±634.70 | 535.96±207.92 |

All data are expressed as means ± standard deviation (n = 5). ^*^ *p* < 0.05, ^**^ *p* < 0.01, ^***^ *p* < 0.001, when compared with the control group. ^#^ *p* < 0.05, ^##^ *p* < 0.01, when compared with groups treated with raw TR at the same dose.

**Table S7**. Laboratory normal range of hematology in Sprague-Dawley rats.

| **TR dose (g/kg)** | By testing agency | By literature  female | By literature |
| --- | --- | --- | --- |
|  |  |  | male |
| WBC(10^9/L) | 3.3-8.7 | 1.13-7.49 | 1.96-8.25 |
| NEUT(%) | 3.3-26.6 | 7.1-33.2 | 6.2-26.7 |
| LYMPH(%) | 68.6-94.5 | 62.2-90 | 66.6-90.3 |
| MONO(%) | 0-4.1 | 0.8-3.9 | 0.8-3.8 |
| EOS(%) | 0-5 | 0.5-4.5 | 0.2-3.5 |
| BASO(%) | 0-1 | 0-0.8 | 0-0.8 |
| LUC(%) | 0-1 | 0.1-0.9 | 0.1-1.1 |
| RBC (10^12/L) | 5.5-9.3 | 7.07-9.03 | 7.27-9.65 |
| HGB(g/L) | 106-156 | 137-168 | 137-176 |
| HCT(%) | 32.7-44.8 | 37.9-49.9 | 39.6-52.5 |
| MCV(fL) | 43.5-62.7 | 49.9-58.3 | 48.9-57.9 |
| MCH(Pg) | 15.8-19.9 | 17.8-20.9 | 17.1-20.4 |
| MCHC(g/L) | 314-360 | 332-379 | 329-375 |
| PLT(10^9/L) | 493-1124 | 680-1200 | 638-1177 |
| PCT(%) | 0.3-0.8 | - | - |
| MPV(fL) | 5.4-9.2 | 6.2-9.8 | 6.2-9.4 |
| PDW(%) | 22.8-87.7 | 42.2-64.4 | 43.2-64.3 |
